# Second Primary Cancer Risks After Breast Cancer in *BRCA1* and *BRCA2* Pathogenic Variant Carriers

**DOI:** 10.1200/JCO.24.01146

**Published:** 2024-10-29

**Authors:** Isaac Allen, Hend Hassan, Yvonne Walburga, Catherine Huntley, Lucy Loong, Tameera Rahman, Sophie Allen, Alice Garrett, Bethany Torr, Andrew Bacon, Craig Knott, Sophie Jose, Sally Vernon, Margreet Lüchtenborg, Joanna Pethick, Francesco Santaniello, Shilpi Goel, Ying-Wen Wang, Katrina Lavelle, Fiona McRonald, Diana Eccles, Eva Morris, Steven Hardy, Clare Turnbull, Marc Tischkowitz, Paul Pharoah, Antonis C. Antoniou

**Affiliations:** 1 Department of Public Health and Primary Care, Centre for Cancer Genetic Epidemiology, University of Cambridge, Cambridge, United Kingdom; 2 National Disease Registration Service, National Health Service England, London, United Kingdom; 3 Division of Genetics and Epidemiology, Institute of Cancer Research, Sutton, United Kingdom; 4 Health Data Insight CIC, Cambridge, United Kingdom; 5 Department of Clinical Genetics, St George’s University Hospitals NHS Foundation Trust, London, United Kingdom; 6 Centre for Cancer, Society and Public Health, Comprehensive Cancer Centre, School of Cancer and Pharmaceutical Sciences, c, London, United Kingdom; 7 Department of Oncology, Hospital of Prato, Azienda USL Toscana Centro, Firenze, Italy; 8 Division of Gynaecologic Oncology, Department of Obstetrics and Gynaecology, Kaohsiung Chang Gung Memorial Hospital, Kaohsiung, Taiwan; 9 Department of Cancer Sciences, Faculty of Medicine, University of Southampton, Southampton, United Kingdom; 10 Health Data Epidemiology Group, Big Data Institute, Nuffield Department of Population Health, University of Oxford, Oxford, United Kingdom; 11 Department of Medical Genetics, Cambridge Biomedical Research Centre, National Institute for Health Research, University of Cambridge, Cambridge, United Kingdom; 12 Department of Computational Biomedicine, Cedars-Sinai Medical Center, Los Angeles, CA

## Abstract

**Purpose:**

Second primary cancer (SPC) risks after breast cancer (BC) in *BRCA1/BRCA2* pathogenic variant (PV) carriers are uncertain. We estimated relative and absolute risks using a novel linkage of genetic testing data to population-scale National Disease Registration Service and Hospital Episode Statistics electronic health records.

**Methods:**

We followed 25,811 females and 480 males diagnosed with BC and tested for germline *BRCA1/BRCA2* PVs in NHS Clinical Genetics centers in England between 1995 and 2019 until SPC diagnosis, death, migration, contralateral breast/ovarian surgery plus 1 year, or the 31st of December 2020. We estimated standardized incidence ratios (SIRs) using English population incidences, hazard ratios (HRs) comparing carriers to noncarriers using Cox regression, and Kaplan-Meier 10-year cumulative risks.

**Results:**

There were 1,840 *BRCA1* and 1,750 *BRCA2* female PV carriers. Compared with population incidences, *BRCA1* carriers had elevated contralateral BC (CBC; SIR, 15.6 [95% CI, 11.8 to 20.2]), ovarian (SIR, 44.0 [95% CI, 31.4 to 59.9]), combined nonbreast/ovarian (SIR, 2.18 [95% CI, 1.59 to 2.92]), colorectal (SIR, 4.80 [95% CI, 2.62 to 8.05]), and endometrial (SIR, 2.92 [95% CI, 1.07 to 6.35]) SPC risks. *BRCA2* carriers had elevated CBC (SIR, 7.70 [95% CI, 5.45 to 10.6]), ovarian (SIR, 16.8 [95% CI, 10.3 to 26.0]), pancreatic (SIR, 5.42 [95% CI, 2.09 to 12.5]), and combined nonbreast/ovarian (SIR, 1.68 [95% CI, 1.24 to 2.23]) SPC risks. Compared with females without *BRCA1/BRCA2* PVs on testing, *BRCA1* carriers had elevated CBC (HR, 3.60 [95% CI, 2.65 to 4.90]), ovarian (HR, 33.0 [95% CI, 19.1 to 57.1]), combined nonbreast/ovarian (HR, 1.45 [95% CI, 1.05 to 2.01]), and colorectal (HR, 2.93 [95% CI, 1.53 to 5.62]) SPC risks. *BRCA2* carriers had elevated CBC (HR, 2.40 [95% CI, 1.70 to 3.40]), ovarian (HR, 12.0 [95% CI, 6.70 to 21.5]), and pancreatic (HR, 3.56 [95% CI, 1.34 to 9.48]) SPC risks. Ten-year cumulative CBC, ovarian, and combined nonbreast/ovarian cancer risks were 16%/6.3%/7.8% (BRCA1 carriers), 12%/3.0%/6.2% (BRCA2 carriers), and 3.6%/0.4%/4.9% (noncarriers). Male *BRCA2* carriers had higher CBC (HR, 13.1 [95% CI, 1.19 to 146]) and prostate (HR, 5.61 [95% CI, 1.96 to 16.0]) SPC risks than noncarriers.

**Conclusion:**

Survivors of BC carrying *BRCA1* and *BRCA2* PVs are at high SPC risk. They may benefit from enhanced surveillance and risk-reduction measures.

## Introduction

*BRCA1* and *BRCA2* pathogenic variant (PV) prevalences in females diagnosed with breast cancer (BC) have been estimated as 1.1% and 1.5%, respectively.^1^ Survivors of BC found to carry *BRCA1* and *BRCA2* PVs are likely to increase in number because of the increasing frequency of genetic testing in oncology^2^ and good survival outcomes, with 15-year BC-specific survival rates estimated as around 81% in *BRCA1* and 75% in *BRCA2* PV carriers.^3^ Second primary cancer (SPC) risks for *BRCA1* and *BRCA2* PV carriers remain uncertain. Studies reporting nonbreast^4–6^ or contralateral BC (CBC) risks^3,6–8^ are limited in number and size. Precise estimates could inform cancer surveillance and risk-reduction options for survivors of BC carrying *BRCA1/BRCA2* PVs. Although male BC is rare,^9^ *BRCA2* PV prevalence among male BC patients is high (8.1%).^10^ To our knowledge, no study has estimated SPC risks after BC in male PV carriers.

We performed a novel linkage of the National Cancer Registration data set (NCRD),^11^ Hospital Episode Statistics Admitted Patient Care (HES APC)^12^ and outpatients (HES OP)^13^ data sets, and individual-level germline testing information from regional molecular genetics laboratories across England^14^ (henceforth germline testing data set). We describe these data sets in the Data Supplement (online only). We established a cohort of individuals diagnosed with BC and tested for *BRCA1* or *BRCA2* PVs through NHS Clinical Genetics centers in England. We estimated relative and absolute SPC risks at combined and specific sites for *BRCA1/BRCA2* PV carriers after a BC diagnosis. We investigated how these risks varied by age at diagnosis, and estrogen receptor (ER) status, of the first BC.

## Methods

### Study Population

We constructed the retrospective cohort using data on all individuals diagnosed with invasive, nonmetastatic BC between January 1, 1995, and December 31, 2019, in England, linked to *BRCA1/BRCA2* PV germline testing data submitted by 16 National Health Service (NHS) molecular genetic laboratories in England. Testing eligibility was based on established guidelines for the same period.^15^ Cohort eligibility was restricted to those with genetic testing information. Surgery data were extracted from the HES APC/OP data sets. Data on death, cancer diagnoses, sex, sociodemographic factors, treatments, and embarkation were drawn from the NCRD. Data on genes tested, pathogenic classes, test dates, coding DNA sequence changes and protein impact of variants, and genetic test free-text records were drawn from the germline testing data set. Pseudonymized patient data were linked using unique tumor and patient identifiers. Consent from patients was not required as these data are collected by NHS England under Section 254 of the Health and Social Care Act 2012. Ethical approval for the data analyses was granted to the CanGene-CanVar research program (REC:18/WS/0192).

### Defining *BRCA1* and *BRCA2* PV Carrier Status

We divided the cohort into *BRCA1* PV carriers, *BRCA2* PV carriers, *BRCA1/BRCA2* PV noncarriers, and those of other *BRCA1/BRCA2* PV status (Data Supplement, Table S1). Survivors of BC were predominantly assigned other carrier status because of being untested for PVs in one of the genes or missing test results (Data Supplement). We do not present analyses for this group unless stated otherwise.

### Statistical Analyses

Follow-up (FU) began at the latest of the *BRCA1* PV test date, *BRCA2* PV test date, and 365 days after the BC diagnosis, and continued until the next cancer diagnosis, death, migration, contralateral breast/ovarian surgery plus 1 year (Data Supplement, Table S2), or the 31st of December 2020. We did not consider cancers diagnosed from death certificates, ipsilateral BCs, CBCs diagnosed <93 days after the first BC, and nonmelanoma skin cancers as SPCs, so FU continued after these diagnoses when applicable. We defined cancer sites using the International Classification of Diseases-10 code groups employed by Cancer Research UK^16^ (Data Supplement, Table S3).

### Comparison of SPC Risks for PV Carriers Relative to Population Risks

To compare cancer incidences in *BRCA1/BRCA2* PV carriers after BC to population incidences, we estimated ratios of observed to expected SPCs (standardized incidence ratios [SIRs]) separately by *BRCA1/BRCA2* PV carrier status for SPCs at the contralateral breast, ovary, all nonbreast/ovarian sites combined, and any other site where at least three cancers were observed in *BRCA1* or *BRCA2 PV* carriers. The expected counts were calculated using age-, calendar year−, sex-, and site-specific incidence rates for the English population,^17^ who predominantly had no cancer history. We filtered cancers diagnosed from death certificates and nonmelanoma skin cancers from the expected counts. In females, we stratified SIRs by age at BC diagnosis (younger than 45 years/45 years or older) and first BC ER status (positive/negative), as both are associated with *BRCA1/BRCA2* PV carrier status^8,18^ and SPC risks.^19^

### Comparison of SPC Risks for *BRCA1* and *BRCA2* PV Carriers Relative to Noncarriers

SIRs estimate SPC risks in survivors of BC carrying *BRCA1/BRCA2* PVs relative to the general population. Therefore, they reflect risk alterations conferred by the first BC, *BRCA1/BRCA2* PVs, and genetic testing selection criteria such as cancer family history (FH).^20^ To compare SPC risks in survivors of BC carrying PVs with survivors of BC tested negative for PVs in both genes, we estimated hazard ratios (HRs) for SPCs at all sites with significantly elevated SIR estimates for *BRCA1* or *BRCA2* carriers, using Cox proportional hazards models. For females, we adjusted these models for age and calendar year at BC diagnosis and ER status of the first BC, where missing ER status data were imputed using multiple imputation by chained equations^21^ (Data Supplement). As a sensitivity analysis, we further adjusted these models for receipt of chemotherapy, radiotherapy, and hormonal therapy. As a separate sensitivity analysis, we included females untested for a PV in one gene and confirmed not to carry a PV in the other gene after predictive testing in the noncarrier group rather than the other carrier group. We performed these sensitivity analyses when estimating HRs for CBC, ovarian cancer (OC), and nonbreast/ovarian cancer, but not for other SPCs because of low event counts. For males, we included only PV carrier status (*BRCA2 PV* carrier or *BRCA1* and *BRCA2* PV noncarrier) in the models because of low sample sizes. To assess whether the effect of a *BRCA1* PV on CBC risk was modified by age at first BC diagnosis in females, we fit a Cox model including an interaction term between continuous age at first BC diagnosis and PV carrier status (separately for *BRCA1* and *BRCA2*) and compared this to the corresponding original model by performing likelihood ratio tests in each imputed data set and comparing the pooled test statistic to an F-distribution.^21^ We tested whether the effect of *BRCA1* or *BRCA2* PVs on CBC, OC, and combined nonbreast/ovarian cancer risks were modified by age at first BC diagnosis, year at first BC diagnosis, and first BC ER status in females analogously. We assessed the proportional hazards assumption by inspecting transformed survival functions (Data Supplement, Figs S9-S11).

### Incidence Rates and Cumulative Risks

In females, we estimated 10-year cumulative CBC, OC, and combined nonbreast/ovarian SPC risks using Kaplan-Meier techniques. We estimated incidences per 10,000 person-years for these cancers between 0-5 years and 5-10 years of FU. We also estimated the corresponding incidences during a 5-year FU period, stratified by year at first BC diagnosis (before 2013/2013 or after). All analyses were stratified by carrier status.

We conducted all analyses in R version 4.3.1 ^22^ (packages in the Data Supplement).

## Results

Unless stated otherwise, results refer to females.

### Cohort Description

**FIG 1:**
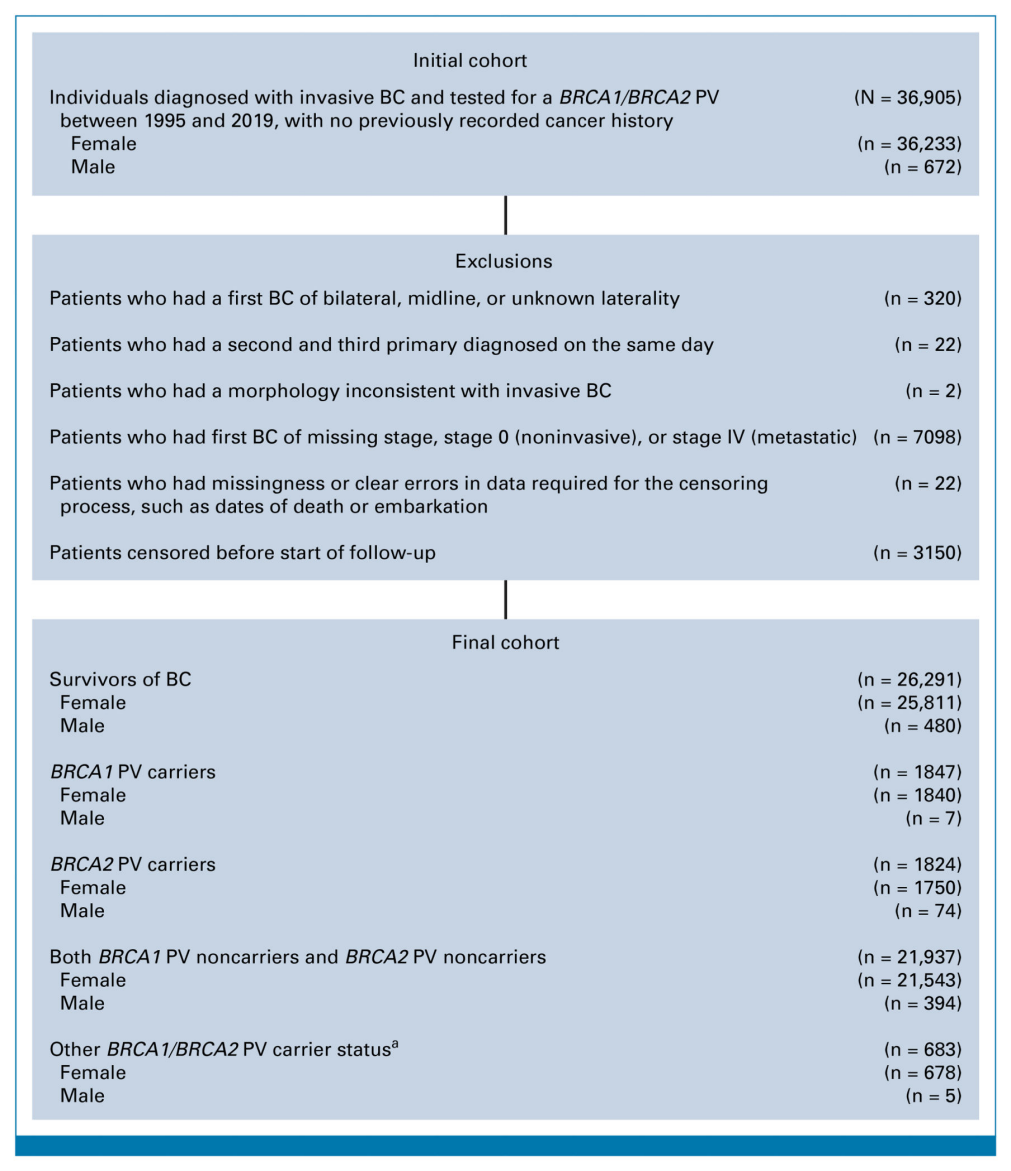
Cohort assembly. ^a^The other *BRCA1/BRCA2* PV carrier status is defined in the Data Supplement (Table S1). BC, breast cancer; FU, follow-up; PV, pathogenic variant. respectively. Further descriptives are in the Data Supplement (Tables S5 and S6 and S13 and Figs S5-S8).

The cohort included 1,840 *BRCA1* PV carriers, 1,750 *BRCA2* PV carriers, and 21,543 noncarriers (Fig 1). Median age at first BC diagnosis was 39 years (IQR, 14 years) in *BRCA1* carriers, 45 years (IQR, 14 years) in *BRCA2* PV carriers, and 46 years (IQR, 15 years) in noncarriers. Corresponding median FU lengths were 3.5 years (IQR, 4.4 years), 3.8 years (IQR, 4.3 years), and 3.5 years (IQR, 3.8 years). CBC was the commonest cancer in all groups (*BRCA1 PV* carriers: 66 events, *BRCA2* PV carriers: 43 events, noncarriers: 237 events). The cohort was primarily of White ethnicity (*BRCA1* PV carriers: 82%, *BRCA2* PV carriers: 90%, noncarriers: 87%). Among those with available ER status data, 71% of *BRCA1* PV carriers, 26% of *BRCA2* PV carriers, and 38% of noncarriers had ER-negative first BC. The majority of the cohort received chemotherapy (*BRCA1* PV carriers: 81%, *BRCA2* PV carriers: 68%, noncarriers: 64%) and radiotherapy (*BRCA1* PV carriers: 52%, *BRCA2* PV carriers: 55%, noncarriers: 66%) and did not receive hormonal therapy (*BRCA1* PV carriers: 86%, *BRCA2* PV carriers: 70%, noncarriers: 74%) by 1 year after BC diagnosis. By the end of FU, most *BRCA1/BRCA2* PV carriers had received contralateral breast surgery (*BRCA1* PV carriers: 64%, *BRCA2* PV carriers: 61%, noncarriers: 22%) and bilateral ovarian surgery (*BRCA1* PV carriers: 55%, *BRCA2* PV carriers: 62%, noncarriers: 10%). Further descriptives are in Table 1 and the Data Supplement (Tables S4 and S12 and Figs S1-S4).

Among males, there were seven *BRCA1* PV carriers, 74 *BRCA2* PV carriers, and 394 noncarriers. They had 0, 15, and 23 SPCs, respectively. Further descriptives are in the Data Supplement (Tables S5 and S6 and S13 and Figs S5-S8).

### Comparison of SPC Risks for PV Carriers Relative to Population Risks

Compared with population-level incidences, PV carriers were at elevated CBC (*BRCA1*: SIR, 15.6 [95% CI, 11.8 to 20.2]; *BRCA2*: SIR, 7.70 [95% CI, 5.45 to 10.6]) and OC (*BRCA1*: SIR, 44.0 [95% CI, 31.4 to 59.9]; *BRCA2*: SIR, 16.8 [95% CI, 10.3 to 26.0]) risks. The magnitudes of both increases were higher in *BRCA1* than *BRCA2* PV carriers (Table 2). *BRCA1/BRCA2* PV carriers had elevated combined nonbreast/ovarian cancer SIRs (*BRCA1*: SIR, 2.18 [95% CI, 1.59 to 2.92]; *BRCA2*: SIR, 1.68 [95% CI, 1.24 to 2.23]). Colorectal (SIR, 4.80 [95% CI, 2.62 to 8.05]) and endometrial (SIR, 2.92 [95% CI, 1.07 to 6.35]) cancer SIRs were increased in *BRCA1* PV carriers. The pancreatic cancer SIR was elevated in *BRCA2* PV carriers (SIR, 5.72 [95% CI, 2.09 to 12.5]).

The CBC SIR was higher in *BRCA1* PV carriers first diagnosed with BC at under age 45 years than at 45 years or over (under age 45 years: SIR, 23.5 [95% CI, 16.6 to 32.3]; 45 years or over: SIR, 9.31 [95% CI, 5.60 to 14.5]). There was no clear difference in CBC SIRs by age at first BC diagnosis in *BRCA2* PV carriers, although SIRs were elevated in both groups (under age 45 years: SIR, 9.58 [95% CI, 5.10 to 16.4]; 45 years or over: SIR, 6.99 [95% CI, 4.52 to 10.3]). There were no clear differences by age at first BC diagnosis in SPC SIRs at other sites in *BRCA1/BRCA2* PV carriers.

Noncarriers had elevated CBC and nonbreast/ovarian cancer SIRs, which were lower than the corresponding *BRCA1*-or *BRCA2*-specific SIRs (CBC: SIR, 3.03 [95% CI, 2.67 to 3.43]; nonbreast/ovarian: SIR, 1.26 [95% CI, 1.14 to 1.38]). The CBC SIR was more elevated in those diagnosed with BC at under age 45 years (under age 45 years: SIR, 4.50 [95% CI, 3.70 to 5.41]; 45 years or over: SIR, 2.43 [95% CI, 2.05 to 2.86]).

There was a modest increased endometrial cancer SIR in noncarriers (SIR, 1.43 [95% CI, 1.06 to 1.89]), and no significant evidence for increased ovarian, colorectal, or pancreatic cancer SIRs. There was some evidence for a nonbreast/ovarian cancer risk difference by age at first BC diagnosis in noncarriers (under 45 years: SIR, 1.68 [95% CI, 1.39 to 2.01]; 45 years or over: SIR, 1.15 [95% CI, 1.02 to 1.28]), which we did not observe for *BRCA1/BRCA2* PV carriers.

We observed no clear SPC SIR differences by first BC ER status at any site, in any carrier group.

Male *BRCA2* PV carriers had elevated CBC (SIR, 431 [95% CI, 48.5 to 1,559]), pancreatic (SIR, 20.2 [95% CI, 4.07 to 59.1]), and prostate (SIR, 4.46 [95% CI, 1.79 to 9.19]) cancer SIRs. No SIRs were significantly elevated in noncarriers (Data Supplement, Table S7).

### Comparison of SPC Risks Between PV Carriers and Noncarriers

*BRCA1* PV carriers were at increased CBC (HR, 3.60 [95% CI, 2.65 to 4.90]), OC (HR, 33.0 [95% CI, 19.1 to 57.1]), colorectal (HR, 2.93 [95% CI, 1.53 to 5.62]), and nonbreast/ovarian (HR, 1.45 [95% CI, 1.05 to 2.01]) cancer risks compared with noncarriers (Table 3). *BRCA2* PV carriers were at increased CBC (HR, 2.40 [95% CI, 1.70 to 3.40]), ovarian (HR, 12.0 [95% CI, 6.70 to 21.5]), and pancreatic (HR, 3.56 [95% CI, 1.34 to 9.48]) cancer risks. There was no significant evidence for interactions between age at diagnosis, year at diagnosis, or ER status of the first BC with *BRCA1* or *BRCA2* PV carrier status when evaluating associations with CBC, OC, or non-breast/ovarian cancer risks. CBC, OC, and nonbreast/ovarian cancer HRs remained similar after adjusting for chemotherapy, radiotherapy, and hormonal therapy (Data Supplement, Table S9), and after including females that tested negative for a PV in one gene after predictive testing, and were untested for PVs in the other gene, in the *BRCA1/BRCA2* PV noncarrier group (Data Supplement, Table S10).

Male *BRCA2* PV carriers had higher CBC and prostate SPC risks than *BRCA1/BRCA2* PV noncarriers (CBC: HR, 13.1 [95% CI, 1.19 to 146]; prostate: HR, 5.61 [95% CI, 1.96 to 16.0]; Data Supplement, Table S8).

### Incidence Rates and Cumulative Risks

**FIG 2:**
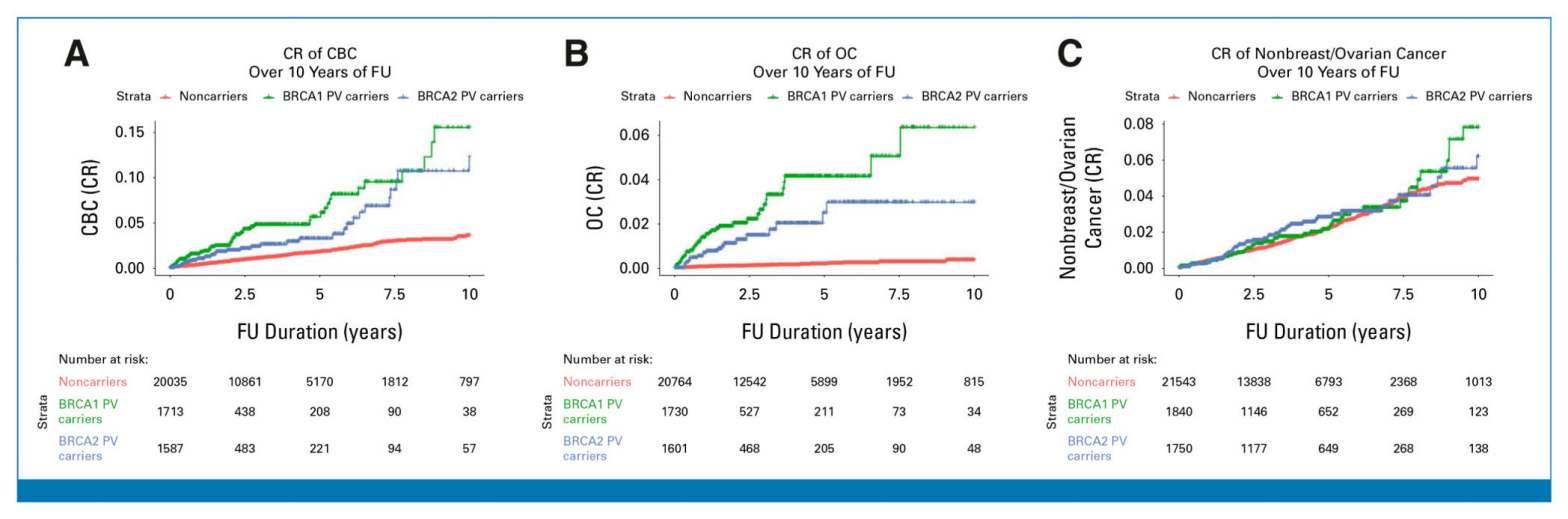
Ten-year cumulative second primary cancer risk curves, stratified by *BRCA1* and *BRCA2* PV carrier status: (A) CBC, (B) OC, and (C) nonbreast/ovarian cancer. CBC, contralateral breast cancer; CR, cumulative risk; FU, follow-up; OC, ovarian cancer; PV, pathogenic variant.

The 10-year cumulative CBC risks were 16% (95% CI, 8.7 to 22) in *BRCA1* PV carriers, 12% (95% CI, 6.5 to 18) in *BRCA2* PV carriers, and 3.6% (95% CI, 2.9 to 4.2) in noncarriers. The corresponding OC and combined nonbreast/ovarian SPC risks were 6.3% (95% CI, 2.8 to 9.7), 3.0% (95% CI, 1.3 to 4.6), and 0.4% (95% CI, 0.1 to 0.6), and 7.8% (95% CI, 4.6 to 11), 6.2% (95% CI, 3.6 to 8.7), and 4.9% (95% CI, 4.2 to 5.6). Ten-year cumulative risk (CR) and incidence estimates are presented in Table 4, with 10-year Kaplan-Meier curves provided in Figure 2.

Within each carrier group, the incidence estimates during a 5-year period for CBC, OC, and nonbreast/ovarian cancer were somewhat higher for those diagnosed with their first BC before 2013 than those diagnosed in 2013 or later (Data Supplement, Table S11).

## Discussion

To our knowledge, this study is one of the first to examine nonbreast cancer risks^4–6^ and one of the largest to examine CBC risks^3,7,8^ after BC in female *BRCA1/BRCA2* PV carriers. It is also the first, to our knowledge, to investigate associations between germline pathogenic variation and SPC risks after male BC. To our knowledge, it is the first study based on a linkage of germline testing laboratory data to population-scale electronic health records (EHRs), minimizing selection biases common in recruitment-based cohort studies.^23^ It is based on very high-quality registry data.^11–14^ This work offers proof of principle that linkages of genetic testing laboratory data to population-scale EHRs allow estimation of understudied cancer risks in novel cohorts.

In females, we found elevated CBC, ovarian, and nonbreast/ovarian SPC risks in *BRCA1/BRCA2* PV carriers, colorectal and endometrial SPC risks in *BRCA1* PV carriers, and pancreatic SPC risks in *BRCA2* PV carriers, relative to the general English population, as measured by the SIRs. These increased SIRs cannot be fully attributed to *BRCA1/BRCA2* PVs as some of the increase will reflect the effect of cancer risk factors associated with having survived a first BC, such as common genetic variation^24,25^ and nongenetic factors such as treatment effects.^19,26^ The ascertainment process will also partly explain the elevated SIRs, as those tested for *BRCA1/BRCA2* PVs are typically highly selected on the basis of criteria such as cancer FH.^20^ Nevertheless, the *BRCA1/BRCA2* SIR estimates were much higher than the corresponding SIRs for noncarriers, and the HR estimates comparing carriers and noncarriers were elevated at most sites with increased SIR estimates. Since the carrier and noncarrier groups in the HR estimations were ascertained in similar fashions and composed of survivors of BC, the effects of the ascertainment process and BC-associated SPC risk factors will likely be attenuated when comparing carriers with noncarriers. This suggests that much of the excess SPC risks are attributable to *BRCA1/BRCA2* PVs. However, the HR estimates may be biased if cancer FH differs between carriers and noncarriers in this cohort. Unfortunately, cancer FH data were unavailable. Notably, the female CBC HR estimates for both *BRCA1* and *BRCA2* PV carriers were consistent with two recent cohort studies.^3,7^

We found higher CBC SIRs for female *BRCA1* PV carriers younger than 45 years at first BC diagnosis compared with those diagnosed when older. This is consistent with population-level observations^19^ and could be explained by the higher proportion of ER-negative BC^18,19^ or more extensive BC FH^26,27^ in *BRCA1* PV carriers younger at BC diagnosis. We found no other notable SIR differences by age at first BC diagnosis in *BRCA1/BRCA2* PV carriers.

The 10-year cumulative CBC, OC, and nonbreast/ovarian cancer risk estimates are applicable to carriers and tested noncarriers ascertained through clinical genetics centers, and the CBC risk estimates for *BRCA1/BRCA2* PV carriers were broadly consistent with a large previous study with similar ascertainment criteria.^8^ However, they would overestimate the risks in *BRCA1/BRCA2* PV carriers unselected for cancer FH, emphasizing the importance of integrating FH in the counseling and risk estimation process.^20^

The male *BRCA2* PV carrier CBC SIR was greater than the corresponding HR, indicating that FH may partly account for the elevated risk, consistent with previous research.^28^ The prostate cancer SIR was consistent with previous research,^29^ and similar to the corresponding HR.

The SIR, HR, and CR estimates in *BRCA1/BRCA2* PV carriers may be inflated by surveillance bias, as cancer surveillance may be heightened after a positive *BRCA1/BRCA2* PV test.^20^ The SIR estimates may be additionally prone to such bias owing to heightened surveillance in survivors of BC relative to the general population.^20^ In addition, the low nonbreast/ovarian/prostate SPC counts may mean some analyses were underpowered, particularly in males. Furthermore, the median FU of under 4 years and median age of 46 years at first BC diagnosis may have precluded the identification of associations with later-or older-onset cancers. Finally, since the criteria for a genetic testing referral changed in 2013,^30^ the influence of FH on the estimates may differ between those tested for BRCA1/2 PVs before 2013 and in 2013 or later. Analyses were adjusted for first BC diagnosis year when estimating HRs and SIRs. However, the absolute incidence estimates were somewhat higher for those diagnosed before 2013 than those diagnosed in 2013 or later. This may also reflect improvements in clinical management over time (Data Supplement Table S11).

The elevated CBC/OC cancer risks, together with previous results,^3,5,7,8^ suggest that females found to carry *BRCA1/BRCA2* PVs may wish to consider risk-reducing options such as contralateral mastectomy and risk-reducing bilateral salpingo-oophorectomy after BC. These recommendations are consistent with results from previous studies.^31,32^

We also found increased CBC and prostate cancer risks in male *BRCA2* PV carriers and elevated colorectal and pancreatic cancer risks in female *BRCA1* and *BRCA2* PV carriers. Although these results were based on low SPC counts, previous findings of elevated first primary risks at the breast and prostate in male *BRCA2* PV carriers, colorectal cancer in female *BRCA1* PV carriers, and pancreatic cancer in female *BRCA2* PV carriers^33^ suggest these associations may be true.

In conclusion, we estimated combined and site-specific relative and absolute SPC risks in BRCA1/2 PV carriers after BC. We investigated risk variability by age at diagnosis and ER status of the first BC in females. This study demonstrates the value of population-scale EHR linkages, and that survivors of BC carrying *BRCA1/BRCA2* PVs are at elevated cancer risks.

## Supplementary Material

Data sharing statement

Supplementary file

## Figures and Tables

**Table 1:** Cohort Description—Age at BC Diagnosis, Years of FU, First BC Diagnosis Dates, Genetic Test Dates, Sociodemographic Factors, and SPC Counts in Females

*BRCA1* PV Carriers		*BRCA2* PV Carriers		*BRCA1/BRCA2* PV Noncarriers		
Median age at BC dx: 39 years (IQR, 14 years)		Median age at BC dx: 45 years (IQR, 14 years)		Median age at BC dx: 46 years (IQR, 15 years)		
Median FU contributed: 3.5 years (IQR, 4.4 years)		Median FU contributed: 3.8 years (IQR, 4.3 years)		Median FU contributed: 3.5 years (IQR, 3.8 years)		
Variable	Entire Cohort, No. (%)	With SPC, No. (%)	Entire Cohort, No. (%)	With SPC, No. (%)	Entire Cohort, No. (%)	With SPC, No. (%)
Age at first BC dx						
Under 45 years	1,237 (67.2)	84 (53.2)	859 (49.1)	30 (26.5)	9,616 (44.6)	245 (33.1)
45 years or over	603 (32.8)	74 (46.8)	891 (50.9)	83 (73.5)	11,927 (55.4)	496 (66.9)
FU contributed						
Under 5 years	1,188 (64.6)	118 (74.7)	1,101 (62.9)	84 (74.3)	14,750 (68.5)	551 (74.4)
5 years or over	652 (35.4)	40 (25.3)	649 (37.1)	29 (25.7)	6,793 (31.5)	190 (25.6)
Year of first BC dx						
1995-1999	99 (5.4)	18 (11.4)	116 (6.6)	21 (18.6)	1,142 (5.3)	104 (14.0)
2000-2004	141 (7.7)	33 (20.9)	154 (8.8)	22 (19.5)	1,324 (6.1)	111 (15.0)
2005-2009	152 (8.3)	26 (16.5)	166 (9.5)	17 (15.0)	1,680 (7.8)	106 (14.3)
2010-2014	590 (32.1)	51 (32.3)	536 (30.6)	34 (30.1)	6,308 (29.3)	246 (33.2)
2015-2019	858 (46.6)	30 (19.0)	778 (44.5)	19 (16.8)	11,089 (51.5)	174 (23.5)
Year of *BRCA1* PV test						
1995-1999	11 (0.6)	1 (0.6)	2(0.1)	0	4 (<0.1)	0
2000-2004	50 (2.7)	9 (5.7)	13 (0.7)	6 (5.3)	113 (0.5)	27 (3.6)
2005-2009	142 (7.7)	25 (15.8)	79 (4.5)	7 (6.2)	956 (4.4)	113 (15.2)
2010-2014	561 (30.5)	54 (34.2)	349 (19.9)	31 (27.4)	5,219 (24.2)	286 (38.6)
2015-2019	1,076 (58.5)	69 (43.7)	740 (42.3)	24 (21.2)	15,251 (70.8)	315 (42.5)
Untested	0	0	567 (32.4)	45 (39.8)	0	0
Year of *BRCA2* PV test						
1995-1999	0	0	10 (0.6)	3 (2.7)	5 (<0.1)	0
2000-2004	17 (0.9)	1 (0.6)	48 (2.7)	9 (8.0)	113 (0.5)	27 (3.6)
2005-2009	63 (3.4)	11 (7.0)	152 (8.7)	16 (14.2)	958 (4.4)	113 (15.2)
2010-2014	384 (20.9)	38 (24.1)	532 (30.4)	46 (40.7)	5,209 (24.2)	286 (38.6)
2015-2019	851 (46.2)	58 (36.7)	1,008 (57.6)	39 (34.5)	15,258 (70.8)	315 (42.5)
Untested	525 (28.5)	50 (31.6)	0	0	0	0
IMD quintile^a^						
1 (most deprived)	353 (19.2)	23 (14.6)	268 (15.3)	24 (21.2)	2,900 (13.5)	126 (17.0)
2	338 (18.4)	30 (19.0)	329 (18.8)	20 (17.7)	3,735 (17.3)	120 (16.2)
3	349 (19.0)	30 (19.0)	342 (19.5)	20 (17.7)	4,437 (20.6)	163 (22.0)
4	397 (21.6)	38 (24.1)	393 (22.5)	22 (19.5)	5,030 (23.3)	171 (23.1)
5 (least deprived)	403 (21.9)	37 (23.4)	418 (23.9)	27 (23.9)	5,441 (25.3)	161 (21.7)
Ethnicity						
White	1,504 (81.7)	138 (87.3)	1,571 (89.8)	109 (96.5)	18,712 (86.9)	690 (93.1)
Black	62 (3.4)	3(1.9)	28 (1.6)	1 (0.9)	546 (2.5)	15 (2.0)
Chinese	7 (0.4)	1 (0.6)	8 (0.5)	0	73 (0.3)	0
Asian	117 (6.4)	9 (5.7)	42 (2.4)	2(1.8)	763 (3.5)	17 (2.3)
Mixed	24 (1.3)	3 (1.9)	6 (0.3)	0	222 (1.0)	6 (0.8)
Other	53 (2.9)	3 (1.9)	35 (2.0)	0	387 (1.8)	9(1.2)
Data missing	73 (4.0)	1 (0.6)	60 (3.4)	1 (0.9)	840 (3.9)	4 (0.5)
With SPCs^b^						
Contralateral breast	66 (3.6)		43 (2.5)		287 (1.3)	
Ovary	47 (2.6)		22 (1.3)		30 (0.1)	
Colorectum	14 (0.8)		6 (0.3)		63 (0.3)	
Lung	6 (0.3)		8 (0.5)		85 (0.4)	
Endometrium	6 (0.3)		4 (0.2)		49 (0.2)	
Pancreas	2(0.1)		6 (0.3)		19 (<0.1)	
Skin (melanoma)	2(0.1)		5 (0.3)		44 (0.2)	
Head and neck	1 (<0.1)		3 (0.2)		14 (<0.1)	
Totals	1,840 (100.0)	158 (100.0)	1,750 (100.0)	113 (100.0)	21,543 (100.0)	741 (100.0)

Abbreviations: BC, breast cancer; dx, diagnosis; FU, follow-up; IMD, indices of multiple deprivation; PV, pathogenic variant; SPC, second primary cancer.

a Quintile refers to the entire UK population, not to the study cohort.

b We also observed two liver, esophagus, and non-Hodgkin lymphoma cancers, one kidney, leukemia, and thyroid cancer, and five other cancers in *BRCA1* carriers. In addition, we observed two esophagus cancers and myelomas, one non-Hodgkin lymphoma and kidney, liver, and thyroid cancer, and eight other cancers in *BRCA2* carriers.

**Table 2:** SIRs for Second Primary Risks in Females

SPC Site	*BRCA1* PV Carriers		*BRCA2* PV Carriers		*BRCA1/BRCA2* PV Noncarriers	
	SIR (95% CI)	Observed SPCs, No.	SIR (95% CI)	Observed SPCs, No.	SIR (95% CI)	Observed SPCs, No.
Entire cohort						
Contralateralbreast	15.6 (11.8 to 20.2)	57	7.70 (5.45 to 10.6)	38	3.03 (2.67 to 3.43)	257
Ovary	44.0 (31.4 to 59.9)	40	16.8 (10.3 to 26.0)	20	1.22 (0.82 to 1.74)	30
Nonbreast/ovarian	2.18 (1.59 to 2.92)	45	1.68 (1.24 to 2.23)	48	1.26 (1.14 to 1.38)	424
Colorectum	4.80 (2.62 to 8.05)	14	1.40 (0.51 to 3.05)	6	1.23 (0.95 to 1.58)	63
Lung	1.98 (0.72 to 4.30)	6	1.62 (0.70 to 3.20)	8	1.42 (1.13 to 1.75)	85
Endometrium	2.92 (1.07 to 6.35)	6	1.36 (0.37 to 3.48)	4	1.43 (1.06 to 1.89)	49
Pancreas	3.03 (0.34 to 10.9)	2	5.72 (2.09 to 12.5)	6	1.48 (0.89 to 2.31)	19
Skin (melanoma)	0.92 (0.10 to 3.31)	2	1.97 (0.63 to 4.59)	5	1.52 (1.11 to 2.04)	44
Head and neck	1.15 (0.02 to 6.38)	1	2.57 (0.52 to 7.52)	3	0.96 (0.51 to 1.65)	13
Younger than 45 years at first BC diagnosis						
Contralateral breast	23.5 (16.6 to 32.3)	38	9.58 (5.10 to 16.4)	13	4.50 (3.70 to 5.41)	111
Ovary	37.4 (20.9 to 61.7)	15	.	0	1.15 (0.46 to 2.36)	7
Nonbreast/ovarian	2.46 (1.52 to 3.76)	21	1.75 (0.93 to 2.99)	13	1.68 (1.39 to 2.01)	117
Colorectum	6.63 (2.66 to 13.7)	7		0	2.13 (1.28 to 3.33)	19
Lung	2.62 (0.29 to 9.46)	2		0	2.47 (1.44 to 3.95)	17
Endometrium	5.59 (1.50 to 14.3)	4	4.35 (0.88 to 12.7)	3	2.39 (1.34 to 3.95)	15
Pancreas		0	11.0 (1.23 to 39.7)	2	.	0
Skin (melanoma)		0	1.96 (0.22 to 7.08)	2	1.45 (0.79 to 2.44)	14
Head and neck		0	5.89 (0.66 to 21.3)	2	1.28 (0.34 to 3.28)	4
Age 45 years or older at first BC diagnosis								
Contralateralbreast	9.31 (5.60 to 14.5)	19	6.99 (4.52 to 10.3)	25	2.43 (2.05 to 2.86)	146
Ovary	49.1 (31.8 to 72.5)	25	23.7 (14.5 to 36.6)	20	1.24 (0.79 to 1.86)	23
Nonbreast/ovarian	1.99 (1.27 to 2.96)	24	1.66 (1.15 to 2.30)	35	1.15 (1.02 to 1.28)	307
Colorectum	3.76 (1.51 to 7.75)	7	1.80 (0.66 to 3.93)	6	1.04 (0.76 to 1.40)	44
Lung	1.76 (0.47 to 4.51)	4	1.92 (0.83 to 3.78)	8	1.28 (0.99 to 1.62)	68
Endometrium	1.49 (0.17 to 5.39)	2	0.44 (0.01 to 2.47)	1	1.22 (0.84 to 1.70)	34
Pancreas	4.25 (0.48 to 15.3)	2	4.61 (1.24 to 11.8)	4	1.71 (1.03 to 2.66)	19
Skin (melanoma)	2.14 (0.24 to 7.72)	2	1.97 (0.40 to 5.75)	3	1.56 (1.05 to 2.22)	30
Head and neck	2.00 (0.03 to 11.1)	1	1.21 (0.02 to 6.73)	1	0.87 (0.40 to 1.65)	9
ER-positive first BC						
Contralateral breast	9.24 (2.98 to 21.6)	5	4.76 (2.05 to 9.38)	8	2.75 (2.19 to 3.40)	84
Ovary	100 (51.7 to 175)	12	20.1 (8.67 to 39.7)	8	1.19 (0.60 to 2.14)	11
Nonbreast/ovarian	1.28 (0.35 to 3.29)	4	1.63 (0.95 to 2.61)	17	1.26 (1.07 to 1.48)	158
Colorectum	4.60 (0.52 to 16.6)	2	1.96 (0.39 to 5.72)	3	1.07 (0.65 to 1.65)	20
Lung		0	1.78 (0.36 to 5.20)	3	1.24 (0.82 to 1.81)	27
Endometrium		0	.	0	1.34 (0.78 to 2.14)	17
Pancreas		0	8.16 (1.64 to 23.9)	3	2.15 (1.03 to 2.95)	10
Skin (melanoma)		0	4.02 (1.08 to 10.3)	4	2.07 (1.31 to 3.11)	23
Head and neck	7.54 (0.10 to 42.0)	1	2.30 (0.03 to 12.8)	1	1.18 (0.43 to 2.57)	6
ER-negative first BC						
Contralateralbreast	21.3 (13.6 to 31.7)	24	5.93 (1.19 to 17.3)	3	3.66 (2.74 to 4.79)	53
Ovary	37.2 (18.5 to 66.5)	11	15.0 (1.69 to 54.3)	2	1.30 (0.42 to 3.04)	5
Nonbreast/ovarian	1.76 (0.88 to 3.16)	11	1.47 (0.40 to 3.77)	4	1.21 (0.91 to 1.57)	56
Colorectum	5.89 (1.90 to 13.7)	5	2.55 (0.03 to 14.2)	1	1.34 (0.61 to 2.55)	9
Lung	3.81 (0.77 to 11.1)	3		0	2.40 (1.40 to 3.85)	17
Endometrium		0		0	0.43 (0.05 to 1.54)	2
Pancreas		0		0	1.28 (0.14 to 4.62)	2
Skin (melanoma)	2.70 (0.30 to 9.73)	2	3.62 (0.05 to 20.1)	1	0.84 (0.23 to 2.15)	4
Head and neck		0	8.19 (0.11 to 45.5)	1	1.00 (0.11 to 3.62)	2

Abbreviations: BC, breast cancer; ER, estrogen receptor; PV, pathogenic variant; SIR, standardized incidence ratio; SPC, second primary cancer.

**Table 3:** Associations Between *BRCA1/BRCA2* PV Carrier Status and SPC Risks, Adjusted for Age and Calendar Year at First BC Diagnosis, and Estrogen Receptor Status of First BC Diagnosis

Cancer Site and *BRCA1/2* PV Carrier Status	Females, No.	Person-Years	Events, No.	HR (95% CI)
Contralateral breast SPCs				
Noncarriers	20,035	70,434	257	1.00 (reference category)
*BRCA1* PV carriers	1,713	3,737	57	3.60 (2.65 to 4.90)
*BRCA2* PV carriers	1,587	4,015	38	2.40 (1.70 to 3.40)
Ovarian SPCs				
Noncarriers	20,764	79,470	30	1.00 (reference category)
*BRCA1* PV carriers	1,730	4,325	40	33.0 (19.1 to 57.1)
*BRCA2* PV carriers	1,601	4,169	20	12.0 (6.70 to 21.5)
Nonbreast/ovarian SPCs				
Noncarriers	21,543	87,814	424	1.00 (reference category)
*BRCA1* PV carriers	1,840	7,971	45	1.45 (1.05 to 2.01)
*BRCA2* PV carriers	1,750	8,016	48	1.24 (0.92 to 1.68)
ColorectalSPCs				
Noncarriers	21,543	87,814	63	1.00 (reference category)
*BRCA1* PV carriers	1,840	7,971	14	2.93 (1.53 to 5.62)
*BRCA2* PV carriers	1,750	8,016	6	1.06 (0.45 to 2.49)
EndometrialSPCs				
Noncarriers	21,543	87,814	49	1.00 (reference category)
*BRCA1* PV carriers	1,840	7,971	6	1.87 (0.73 to 4.74)
*BRCA2* PV carriers	1,750	8,016	4	0.86 (0.30 to 2.44)
Pancreatic SPCs				
Noncarriers	21,543	87,814	19	1.00 (reference category)
*BRCA1* PV carriers	1,840	7,971	2	1.84 (0.36 to 9.32)
*BRCA2* PV carriers	1,750	8,016	6	3.56 (1.34 to 9.48)

Abbreviations: BC, breast cancer; HR, hazard ratio; PV, pathogenic variant; SPC, second primary cancer.

**Table 4:** Incidence Rates, 10-Year CRs, and Associated Statistics for SPC Risks

FU Time Elapsed	Total Person-Years	BC Survivors, No.	O, No.	Inc (95% CI)	CR (95% CI)
Contralateral breast SPCs in *BRCA1* PV carriers					
<5 years	3,095	1,713	44	142 (105 to 189)	5.6 (3.6 to 7.5)
5-10 years	485	208	11	227 (120 to 393)	16 (8.7 to 22)
Contralateral breast SPCs in *BRCA2* PV carriers					
<5 years	3,249	1,587	26	80.0 (53.5 to 115)	3.2 (1.8 to 4.7)
5-10 years	558	221	11	197 (105 to 341)	12 (6.5 to 18)
Contralateral breast SPCs in *BRCA1/BRCA2* PV noncarriers					
<5 years	57,283	20,035	202	35.3 (30.6 to 40.4)	1.7 (1.5 to 2.0)
5-10 years	11,256	5,170	47	41.8 (31.1 to 55.0)	3.6 (2.9 to 4.2)
Ovarian SPCs in *BRCA1* PV carriers					
<5 years	3,708	1,730	38	102 (73.6 to 139)	4.1 (2.6 to 5.7)
5-10 years	443	211	2	45.1 (8.99 to 145)	6.3 (2.8 to 9.7)
Ovarian SPCs in *BRCA2* PV carriers					
<5 years	3,487	1,601	19	54.5 (33.9 to 83.3)	2.5 (1.1 to 3.9)
5-10 years	529	205	1	18.9 (1.72 to 88.2)	3.0 (1.3 to 4.6)
Ovarian SPCs in *BRCA1/BRCA2* PV noncarriers					
<5 years	65,116	20,764	24	3.69 (2.42 to 5.39)	0.2 (0.1 to 0.2)
5-10 years	12,515	5,899	5	4.03 (1.53 to 8.83)	0.4 (0.1 to 0.6)
Nonbreast/ovarian SPCs in *BRCA1* PV carriers					
<5 years	5,944	1,840	26	43.7 (29.2 to 63.1)	2.1 (1.3 to 3.0)
5-10 years	1,540	652	16	104 (61.8 to 165)	7.8 (4.6 to 11)
Nonbreast/ovarian SPCs in *BRCA2* PV carriers					
<5 years	5,983	1,750	34	56.8 (40.0 to 78.4)	2.8 (1.8 to 3.8)
5-10 years	1,586	649	9	56.7 (28.0 to 104)	6.2 (3.6 to 8.7)
Nonbreast/ovarian SPCs in *BRCA1/BRCA2* PV noncarriers					
<5 years	70,649	21,543	300	42.5 (37.9 to 47.5)	2.2 (1.9 to 2.5)
5-10 years	14,753	6,793	93	63.0 (51.2 to 76.9)	4.9 (4.2 to 5.6)

Abbreviations: BC, breast cancer; CR, cumulative risk; FU, follow-up; Inc, incidence per 10,000 person-years; O, second primaries observed; PV, pathogenic variant; SPC, second primary cancer.

## Data Availability

A data sharing statement provided by the authors is available with this article at DOI https://doi.org/10.1200/JCO.24.01146.

## Context

### Key Objective

Second primary cancer (SPC) risks after breast cancer (BC) in *BRCA1/BRCA2* pathogenic variant (PV) carriers are unclear. This study investigates associations between *BRCA1/BRCA2* PVs and SPCs in a novel, population-scale linkage of electronic health records from the National Health Service England and genetic testing data from clinical laboratories across England.

### Knowledge Generated

Elevated risks were found for SPCs at the contralateral breast/ovary in female *BRCA1/BRCA2* PV carriers, colorectum in female *BRCA1* PV carriers, pancreas in female *BRCA2* PV carriers, and contralateral breast/prostate in male *BRCA2* PV carriers. Risks were particularly elevated in females younger at first BC diagnosis.

### Relevance (*K.D. Miller*)

Patients found to have PV in *BRCA1/BRCA2* face difficult decisions including considering prophylactic surgery and enhanced screening. The more detailed information on second cancer risks provided by this analysis facilitates crucial shared decision making.*

*Relevance section written by JCO Senior Deputy Editor Kathy D. Miller, MD.
