# Supplementary material for: Second Primary Cancer Risks After Breast Cancer in BRCA1 and BRCA2 Pathogenic Variant Carriers
Source: J Clin Oncol. Author manuscript; Available in PMC 2025 Feb 20. (PMC7616773; doi:10.1200/JCO.24.01146)
Supplement: Data sharing statement [file EMS199754-supplement-Data_sharing_statement.pdf]

Data Supplement

Methods

Data sources

The NCRD and HES APC/OP datasets have been comprehensively described previously (11–13), and were summarized in a prior analysis of SPC risks in the general English population of BC survivors (19). Briefly, the National Cancer Registration and Analysis Service, as part of NHSE, uses the NHS number (a unique identifier across the English healthcare system), dates of birth, and full names and addresses to link comprehensive data on around 300,000 malignant tumours diagnosed annually in England. The HES APC dataset contains data relevant to hospital stays, including dates of admission and discharge and procedure records. The HES OP dataset contains individual-level records of outpatient care, such as appointment dates and procedures performed. The three datasets have respectively collected data since 1971, 1989, and 2003. However, these datasets have incomplete data from private healthcare providers that do not receive NHS funding. This is estimated to compose 1-2% of hospital activity in England (12).

The clinical genetic testing dataset was formed by collecting pseudonymised data submissions of *BRCA1* and *BRCA2* germline genetic testing data from the 16 English NHS clinical genetics laboratories that conduct this testing. No restrictions were placed on the format of the data submissions. Algorithms were developed to extract data from the data submissions into a common data model within the National Disease Registration Service. The amalgamated data were linked to the NCRD using pseudonyms (reproducibly encrypted identifiers based on NHS number, postcode and date of birth). The methods used to collect, extract, quality assure and link *BRCA1/BRCA2* clinical genetic testing data to the NCRD in this analysis are the same as those employed to amalgamate and link MMR gene clinical genetic testing data by Loong et al. in their recent publication (14), which also contains more detailed descriptions of the methods and the common data model for reference.

Defining *BRCA1* and *BRCA2* pathogenic variant carrier status

Data on BC survivors first diagnosed with non-metastatic and non-invasive BC between 1995 and 2019 were identified in the NCRD and were linked to the genetic testing dataset using unique patient identifiers (14). BC survivors identified in the NCRD with no linkage to the germline data were considered to not have received a *BRCA1* or *BRCA2* germline test and were not included in this study.

We developed a pipeline to classify the pathogenicity of single nucleotide variants (SNVs) and insertions and deletions (InDels). Automated classifications were generated based on the American College for Medical Genetics criteria for variant classification[S1], with additional guidance from the Cancer Variant Interpretation Group UK[S2], which provided a framework for scoring of individual evidence elements and combining these to create the final classification. For SNVs, evidence was assembled from functional assay results, ribonucleic acid studies, variant frequency in the germline dataset compared to a non-cancer control population in the Genome Aggregation Database, *in silico* predictions of protein effect and splicing impact, previous classifications from ClinVar, and data from genetic epidemiology studies[S3-S22]. For InDels, the pipeline instead utilised an automated application of the PVS1 criterion to classify variants as non-pathogenic, pathogenic, or as variants of uncertain significance (VUS)[S1]. For all variants initially classified as VUS, we manually searched for their protein impact and coding DNA sequence changes in the online tools dbSNP[S23] and VarSome[S24], and updated the classification if a relevant result was found.

We then categorised germline test results into three possible pipeline outputs: “Normal”, “VUS”, and “Pathogenic”. “Normal” included patients without pathogenic variants, with SNVs of class 1 (benign) and 2 (likely benign), or with InDels classified as non-pathogenic. “Pathogenic” included patients with SNVs of class 4 (likely pathogenic) and 5 (pathogenic), small InDels classified as pathogenic, and large InDels or copy number variants (CNVs) identified as protein-truncating or affecting one or more exons. “VUS” included patients with class 3 SNVs, VUS InDels, and other CNVs.

We then combined the pipeline outputs with the following data from the germline testing dataset: genetic test status (normal, abnormal, or missing), original laboratory pathogenicity classifications (where available), and raw laboratory report text records. To define a final pathogenicity status (negative or positive) for each genetic test, we took all tests with an abnormal or missing genetic test status and assigned a positive final pathogenicity status to all such tests where either the original laboratory pathogenicity classification was class 4 (likely pathogenic) or 5 (pathogenic) or the variant

both had been identified as pathogenic by the pipeline described above, and either had an original laboratory classification of class 3 (VUS) or had no pathogenicity class data available from the laboratory. Finally, we identified additional test results with pathogenic exon deletions by using regular expressions to scrape the raw laboratory report text records and defined all corresponding tests as having a positive pathogenicity status. We assigned all remaining tests a negative pathogenicity status.

Following our classifications of each genetic test, we then defined patients as *BRCA1* PV carriers, *BRCA2* PV carriers, *BRCA1* and *BRCA2* PV non-carriers, and other *BRCA1* and *BRCA2* PV carrier status according to Table S1.

Table S1: Definitions of *BRCA1* and *BRCA2* pathogenic variant carrier status

***BRCA1/BRCA2*[1] carrier status**	***BRCA1*[2] PV[3] test result[4]**	***BRCA2*[5] PV test result**
BRCA1 PV carrier	Positive for PV	Negative or untested for PV, or test result missing
BRCA2 PV carrier	Negative or untested for PV, or test result missing	Positive for PV
BRCA1 and BRCA2 PV non-carrier	Negative for PV	Negative for PV
Other BRCA1 and BRCA2 PV carrier status	*BRCA1* PV positive and *BRCA2* PV positive, *BRCA1* PV negative and *BRCA2* PV missing or untested, *BRCA1* PV missing and *BRCA2* PV negative, missing or untested, or *BRCA1* PV untested and *BRCA2* PV negative or missing	

1: BReast CAncer gene 1 and BReast CAncer gene 2, 2: BReast CAncer gene 1, 3: Pathogenic Variant, 4: Possible results: positive, negative, untested, result missing. If a patient had multiple test results for the same gene, then the first positive test was used to define their *BRCA1/BRCA2* carrier status. If there were no positive tests, then the first negative test was used. If all results were missing, then the first test with a missing result was used. 5: BReast CAncer gene 2

We defined patients with both positive *BRCA1* and *BRCA2* test results as having other PV carrier status instead of including these patients in both the *BRCA1* and the *BRCA2* PV carrier groups both to allow for a complete partitioning of the cohort by PV carrier status and because there is some evidence that cancer risks vary in double heterozygotes compared to women with a PV in only one of the *BRCA1* or *BRCA2* genes[S25]. We were unable to examine SPC risks in double heterozygotes separately instead due to insufficient sample size.

Imputation of missing data

We imputed missing data for ER status of the first breast tumour in females using the random forest method with 10 cycles for 10 imputations, using the year of first BC diagnosis, age at first BC diagnosis, ethnicity, IMD quintile, first breast tumour size in centimetres, number of nodes involved in first breast tumour, grade of first breast tumour, morphology of first breast tumour, ER status of first breast tumour, HER2 status of first breast tumour, *BRCA1* PV carrier status, *BRCA2* PV carrier status, vital status (whether patient lived, died, or embarked the UK during follow-up), whether the patient had chemotherapy by one year following the first BC diagnosis, whether the patient had radiotherapy by one year following the first BC diagnosis, whether the patient had hormonal therapy by one year following the first BC diagnosis, whether the patient developed a cancer during follow-up (without censoring at surgeries)[S26], and a Nelson-Aalen estimator for the development of a cancer (without censoring at surgeries)[S26] as predictive variables.

We considered data on individuals defined as having 'Other' *BRCA1/BRCA2* PV carrier status relevant for imputing missing ER status data, and therefore included such data in this imputation process.

There were 26 females whose first BC was recorded as being of 'borderline' ER status. We regarded these patients as having missing data on ER status and imputed as above.

Approach to surgical censoring

Follow-up for CBC and OC was censored at one year following the surgeries in Table S2 unless laterality data for a contralateral breast surgery was missing. Any such surgery performed within a year of the first BC diagnosis was assumed to have been performed on the ipsilateral breast, and therefore we did not include such surgeries at CBC censoring events. We selected the surgeries in Table S2 by extracting the subset of surgeries defined as breast cancer curative in the HES APC and HES OP datasets and selecting the subset of these surgeries we judged likely to have some impact on cancer risks. For example, if a breast surgery would leave the entire contralateral breast in place (such as a biopsy), we would not include it in the group of CBC-censoring surgeries below.

Table S2: Surgeries considered as censoring events for follow-up for second primary contralateral breast cancer and second primary ovarian cancer

Censoring surgeries for second primary contralateral breast cancer	Censoring surgeries for second primary ovarian cancer
Total mastectomy and excision of both pectoral muscles and part of chest wall	Abdominal hysterocolpectomy and excision of periuterine tissue
Total mastectomy and excision of both pectoral muscles NEC[1]	Abdominal hysterectomy and excision of periuterine tissue NEC
Total mastectomy and excision of pectoralis minor muscle	Vaginal hysterocolpectomy and excision of periuterine tissue
Total mastectomy NEC	Bilateral salpingoophorectomy
Subcutaneous mastectomy	Bilateral oophorectomy NEC
Skin sparing mastectomy	Salpingoophorectomy of remaining solitary fallopian tube and ovary
Other specified total excision of breast	Oophorectomy of remaining solitary ovary NEC
Unspecified total excision of breast	Other specified unilateral excision of adnexa of uterus
Quadrantectomy of breast	Unspecified unilateral excision of adnexa of uterus
Partial excision of breast NEC	Salpingoophorectomy NEC
Excision of lesion of breast NEC	Oophorectomy NEC
Re-excision of breast margins	Other specified other open operations on ovary
Wire guided partial excision of breast	Total exenteration of pelvis
Wire guided excision of lesion of breast	Anterior exenteration of pelvis
Other specified other excision of breast	Posterior exenteration of pelvis
Unspecified other excision of breast	Other specified clearance of pelvis
Subareolar excision of mammary duct	Unspecified clearance of pelvis
Excision of mammary duct NEC	..
Excision of lesion of mammary duct	..
Excision of nipple	..
Extirpation of lesion of nipple	..
Capsulectomy of breast	..
Interstitial laser destruction of lesion of breast	..
Other specified destruction of lesion of breast	..
Unspecified destruction of lesion of breast	..

1: Not Elsewhere Specified

Second primary cancer site definitions

We defined cancer sites according to the groupings of ICD-10 codes used by Cancer Research UK (16) (Table S3). We included peritoneal cancers in OC counts to ensure consistency with the World Health Organisation's 2014 revision of the primary site definition [S27, S28].

Table S3: ICD-10 code groups used to define second primary cancer sites

Cancer site	ICD[1]-10 code group
All non-breast and non-ovarian sites combined	C00-C97, excluding C44, C50, C48, C56-57.4
Contralateral breast	C50
Ovary	C48, C56-57.4
Stomach	C16
Pancreas	C25
Colorectum	C18-20
Endometrium	C54-55
Skin (melanoma)	C43
Lung	C33-34
Non-Hodgkin's lymphoma	C82-86
Kidney	C64-66, C68
Blood (leukaemia)	C91-95
Head and neck	C00-14, C30-32
Bladder	C67
Oesophagus	C15
Blood (myeloma)	C90
Liver	C22
Prostate	C61
Brain and central nervous system	C70-72
Thyroid	C73

1: International Classification of Diseases

R packages used

All statistical analyses were performed in R version 4.3.1 (22). We used the packages data.table[S29], DBI[S30], dplyr[S31], epiR[S32], forcats[S33], getPass[S34], mice[S35], lubridate[S36], readxl[S37], rJava[S38], RJDBC[S39], survival[S40], survminer[S41] and svDialogs[S42].

Results

Table S4: Cohort description – first breast cancer pathology and treatments in females

	***BRCA1*[1] PV[2] carriers**		***BRCA2*[3] PV carriers**		***BRCA1/BRCA2*[4] PV non-carriers**	
	**N[5] (entire cohort)**	**N (with SPC[6])**	**N (entire cohort)**	**N (with SPC)**	**N (entire cohort)**	**N (with SPC)**
**Size of first breast tumour (cm[7])**						
<2	592 (32.2)	60 (38.0)	611 (34.9)	38 (33.6)	8010 (37.2)	317 (42.8)
>=2	747 (40.6)	64 (40.5)	780 (44.6)	51 (45.1)	8501 (39.5)	277 (37.4)
Data missing	501 (27.2)	34 (21.5)	359 (20.5)	24 (21.2)	5032 (23.4)	147 (19.8)
**Number of nodes involved in first breast tumour**						
0	701 (38.1)	66 (41.8)	654 (37.4)	36 (31.9)	8658 (40.2)	293 (39.5)
>0	287 (15.6)	19 (12.0)	463 (26.5)	33 (29.2)	4792 (22.2)	174 (23.5)
Data missing	852 (46.3)	73 (46.2)	633 (36.2)	44 (38.9)	8093 (37.6)	274 (37.0)
**Grade of first breast tumour**						
1	21 (1.1)	5 (3.2)	55 (3.1)	5 (4.4)	2023 (9.4)	91 (12.3)
2	220 (12.0)	12 (7.6)	653 (37.3)	49 (43.4)	8165 (37.9)	320 (43.2)
3	1532 (83.3)	132 (83.5)	976 (55.8)	52 (46.0)	10688 (49.6)	297 (40.1)
Data missing	67 (3.6)	9 (5.7)	66 (3.8)	7 (6.2)	667 (3.1)	33 (4.5)
**Morphology of first breast tumour**						
Ductal	1679 (91.2)	144 (91.1)	1498 (85.6)	95 (84.1)	18094 (84.0)	580 (78.3)
Lobular	19 (1.0)	2 (1.3)	135 (7.7)	10 (8.8)	1560 (7.2)	60 (8.1)
Other	142 (7.7)	12 (7.6)	117 (6.7)	8 (7.1)	1889 (8.8)	101 (13.6)
**ER[8] status of first breast tumour**						
Positive	327 (17.8)	22 (13.9)	790 (45.1)	34 (30.1)	8459 (39.3)	261 (35.2)
Negative	812 (44.1)	51 (32.3)	277 (15.8)	9 (8.0)	5219 (24.2)	125 (16.9)
Data missing	701 (38.1)	85 (53.8)	683 (39.0)	70 (61.9)	7865 (36.5)	355 (47.9)

**HER2[9] status of first breast tumour**						
Positive	56 (3.0)	4 (2.5)	107 (6.1)	2 (1.8)	2130 (9.9)	47 (6.3)
Negative	1115 (60.6)	61 (38.6)	921 (52.6)	40 (35.4)	12052 (55.9)	325 (43.9)
Data missing	669 (36.4)	93 (58.9)	722 (41.3)	71 (62.8)	7361 (34.2)	369 (49.8)
**Has had chemotherapy[10]**						
Yes	1496 (81.3)	117 (74.1)	1189 (67.9)	57 (50.4)	13767 (63.9)	394 (53.2)
No	344 (18.7)	41 (25.9)	561 (32.1)	56 (49.6)	7776 (36.1)	347 (46.8)
**Has had radiotherapy[10]**						
Yes	950 (51.6)	102 (64.6)	967 (55.3)	70 (61.9)	14271 (66.2)	510 (68.8)
No	890 (48.4)	56 (35.4)	783 (44.7)	43 (38.1)	7272 (33.8)	231 (31.2)
**Has had hormonal therapy[10]**						
Yes	252 (13.7)	31 (19.6)	520 (29.7)	48 (42.5)	5715 (26.5)	256 (34.5)
No	1588 (86.3)	127 (80.4)	1230 (70.3)	65 (57.5)	15828 (73.5)	485 (65.5)
**Has had contralateral breast surgery[11]**						
Yes	1169 (63.5)	60 (38.0)	1062 (60.7)	35 (31.0)	4712 (21.9)	138 (18.6)
No	671 (36.5)	98 (62.0)	688 (39.3)	78 (69.0)	16831 (78.1)	603 (81.4)
**Has had bilateral ovarian surgery[11]**						
Yes	1013 (55.1)	84 (53.2)	1080 (61.7)	52 (46.0)	2159 (10.0)	80 (10.8)
No	827 (44.9)	74 (46.8)	670 (38.3)	61 (54.0)	19384 (90.0)	661 (89.2)
**Totals**						
..	1840 (100.0)	158 (100.0)	1750 (100.0)	113 (100.0)	21543 (100.0)	741 (100.0)

1: Breast Cancer gene 1, 2: Pathogenic Variant, 3: Breast Cancer gene 2, 4: Breast Cancer gene 1 and Breast Cancer gene 2, 5: Number of breast cancer survivors, 6: Second Primary Cancer, 7: Centimetres, 8: Estrogen Receptor, 9: Human Epidermal Growth Factor 2, 10: By one year following first breast cancer diagnosis, 11: By end of follow-up

Table S5: Cohort description – age at breast cancer diagnosis, years of follow-up, first breast cancer diagnosis dates, genetic test dates, sociodemographic factors, and second primary cancer counts in males

	***BRCA2*[1] PV[2] carriers**		***BRCA1/BRCA2*[3] PV non-carriers**	
	Median age at BC[4] dx[5]: 64y[6] (IQR[7]: 14y)		*Median age at BC dx: 62y (IQR: 18y)*	
	Median FU[8] contributed: 3.0y (IQR: 3.8y)		*Median FU contributed: 3.2y (IQR: 3.7y)*	
	**N[9] (entire cohort)**	**N (with SPC[10])**	**N (entire cohort)**	**N (with SPC)**
**Age at first BC dx**				
Under 60y	25 (33.8)	2 (13.3)	181 (45.9)	6 (26.1)
60y or over	49 (66.2)	13 (86.7)	213 (54.1)	17 (73.9)
**FU contributed**				
Under 5y	49 (66.2)	11 (73.3)	281 (71.3)	18 (78.3)
5y or over	25 (33.8)	4 (26.7)	113 (28.7)	5 (21.7)
**Year of first BC dx**				
2014 or earlier	44 (59.5)	11 (73.3)	188 (47.7)	17 (73.9)
2015-2019	30 (40.5)	4 (26.7)	206 (52.3)	6 (26.1)
**Year of *BRCA1*[11] PV test**				
2014 or earlier	26 (35.1)	8 (53.3)	119 (30.2)	13 (56.5)
2015-2019	36 (48.6)	4 (26.7)	275 (69.8)	10 (43.5)
Untested	12 (16.2)	3 (20.0)	0 (0.0)	0 (0.0)
**Year of *BRCA2* PV test**				
2014 or earlier	32 (43.2)	11 (73.3)	119 (30.2)	13 (56.5)
2015-2019	42 (56.8)	4 (26.7)	275 (69.8)	10 (43.5)
Untested	0 (0.0)	0 (0.0)	0 (0.0)	0 (0.0)

**IMD[12] quintile[13]**				
1 (most deprived)	12 (16.2)	3 (20.0)	51 (12.9)	1 (4.3)
2	7 (9.5)	1 (6.7)	68 (17.3)	6 (26.1)
3	18 (24.3)	3 (20.0)	88 (22.3)	7 (30.4)
4	18 (24.3)	5 (33.3)	92 (23.4)	6 (26.1)
5 (least deprived)	19 (25.7)	3 (20.0)	95 (24.1)	3 (13.0)
**Ethnicity**				
White	64 (86.5)	14 (93.3)	335 (85.0)	23 (100.0)
Black	2 (2.7)	0 (0.0)	7 (1.8)	0 (0.0)
Chinese	0 (0.0)	0 (0.0)	0 (0.0)	0 (0.0)
Asian	3 (4.1)	0 (0.0)	25 (6.3)	0 (0.0)
Mixed	0 (0.0)	0 (0.0)	0 (0.0)	0 (0.0)
Other	0 (0.0)	0 (0.0)	6 (1.5)	0 (0.0)
Data missing	5 (6.8)	1 (6.7)	21 (5.3)	0 (0.0)
**With SPCs[14]**				
Contralateral breast	2 (2.7)	..	1 (0.3)	..
Prostate	7 (9.5)	..	8 (2.0)	..
Pancreas	3 (4.1)	..	0 (0.0)	..
**Totals**				
..	74 (100.0)	15 (100.0)	394 (100.0)	23 (100.0)

1: BReast CAncer gene 2, 2: Pathogenic Variant, 3: BReast CAncer gene 1 and BReast CAncer gene 2, 4: Breast Cancer, 5: Diagnosis, 6: Years, 7: Interquartile Range, 8: Follow-up, 9: Number and percentage of breast cancer survivors, 10: Second Primary Cancer, 11: BReast Cancer gene 1, 12: Indices of Mutiple Deprivation, 13: Quintile refers to the entire UK population, not to the study cohort, 14: We also observed 1 head and neck, stomach, and blood (myeloid leukaemia) cancer in *BRCA1* pathogenic variant carriers and 6 colorectal cancers, 3 oesophageal cancers, 1 lung, kidney, thyroid and blood (non-Hodgkin's lymphoma) cancer, and 1 other cancer in *BRCA1* and *BRCA2* pathogenic variant non-carriers.

Table S6: Cohort description – first breast cancer pathology and treatments in males

	BRCA2[1] PV[2] carriers		*BRCA1/BRCA2*[3] PV non-carriers	
	N[4] (entire cohort)	N (with SPC[5])	N (entire cohort)	N (with SPC)
**Size of first breast tumour (cm[6])**				
<2	35 (47.3)	10 (66.7)	176 (44.7)	4 (17.4)
>=2	36 (48.6)	5 (33.5)	173 (43.9)	15 (65.2)
Data missing	3 (4.1)	0 (0.0)	45 (11.4)	4 (17.4)
**Number of nodes involved in first breast tumour**				
0	25 (33.8)	5 (33.3)	180 (45.7)	9 (39.1)
>0	37 (50.0)	6 (40.0)	136 (34.5)	9 (39.1)
Data missing	12 (16.2)	4 (26.7)	78 (19.8)	5 (21.7)
**Grade of first breast tumour**				
1	0 (0.0)	0 (0.0)	34 (8.6)	4 (17.4)
2	27 (36.5)	5 (33.3)	228 (57.9)	14 (60.9)
3	45 (60.8)	10 (66.7)	116 (29.4)	4 (17.4)
Data missing	2 (2.7)	0 (0.0)	16 (4.1)	1 (4.3)
**Morphology of first breast tumour**				
Ductal	68 (91.9)	14 (93.3)	351 (89.1)	20 (87.0)
Lobular	2 (2.7)	1 (6.7)	4 (1.0)	0 (0.0)
Other	4 (5.4)	0 (0.0)	39 (9.9)	3 (13.0)
**ER[7] status of first breast tumour**				
Positive	56 (75.7)	9 (60.0)	277 (70.3)	13 (56.5)
Negative	1 (1.4)	0 (0.0)	4 (1.0)	10 (43.5)
Data missing	17 (23.0)	6 (40.0)	113 (28.7)	0 (0.0)

**HER2[8] status of first breast tumour**				
Positive	5 (6.8)	0 (0.0)	27 (6.9)	0 (0.0)
Negative	42 (56.8)	8 (53.3)	235 (59.6)	13 (56.5)
Data missing	27 (36.5)	7 (46.7)	132 (33.5)	10 (43.5)
**Has had chemotherapy[9]**				
Yes	39 (52.7)	9 (60.0)	140 (35.5)	8 (34.8)
No	35 (47.3)	6 (40.0)	254 (64.5)	15 (65.2)
**Has had radiotherapy[9]**				
Yes	46 (62.2)	8 (53.3)	192 (48.7)	11 (47.8)
No	28 (37.8)	7 (46.7)	202 (51.3)	12 (52.2)
**Has had hormonal therapy[9]**				
Yes	31 (41.9)	7 (46.7)	166 (42.1)	10 (43.5)
No	43 (58.1)	8 (53.3)	228 (57.9)	13 (56.5)
**Has had contralateral breast surgery[10]**				
Yes	17 (23.0)	1 (6.7)	34 (8.6)	3 (13.0)
No	57 (77.0)	14 (93.3)	360 (91.4)	20 (87.0)
**Totals**				
..	74 (100.0)	15 (100.0)	394 (100.0)	23 (100.0)

1: Breast Cancer gene 2, 2: Pathogenic Variant, 3: Breast Cancer gene 1 and 1: Breast Cancer gene 2, 4: Number of breast cancer survivors, 5: Second Primary Cancer, 6: Centimetres, 7: Estrogen Receptor, 8: Human Epidermal Growth Factor 2, 9: By one year following first breast cancer diagnosis, 10: By end of follow-up

Table S7: Standardized incidence ratios for second primary risks in males

	BRCA2[1] PV[2] carriers		*BRCA1/BRCA2*[3] PV non-carriers	
**SPC[4] site**	**SIR[5] (95% CI[6])**	**Observed SPCs**	**SIR (95% CI)**	**Observed SPCs**
Contralateral breast	431 (48.5-1559)	2	43.5 (0.57-242)	1
Prostate	4.46 (1.79-9.19)	7	1.13 (0.48-2.22)	8
Pancreas	20.2 (4.07-59.1)	3	..	0

1: Breast Cancer gene 2, 2: Pathogenic Variant, 3: Breast Cancer gene 1 and 2, 4: Second Primary Cancer, 5: Standardized Incidence Ratio, 6: Second Primary Cancer

Table S8: Associations between *BRCA1/BRCA2* pathogenic variant carrier status and second primary cancer risks in males

	Number of males	Person Years	Events	HR[1] (95%CI[2])
Contralateral breast SPCs[3]				
Non-carriers	389	1459	1	1.00 (reference category)
BRCA2[4] PV[5] carriers	74	251	2	13.1 (1.19-146)
Prostate SPCs				
Non-carriers	394	1554	8	1.00 (reference category)
BRCA2 PV carriers	74	281	7	5.61 (1.96-16.0)

1: Hazard Ratio, 2: Confidence Interval, 3: Second Primary Cancer, 4: Breast Cancer gene 2, 5: Pathogenic Variant

Note: Although we found an elevated SIR for stomach cancers in male *BRCA2* PV carriers, we could not estimate hazard ratios for stomach cancers as there were no stomach cancers observed in the reference group.

Table S9: Associations between *BRCA1/BRCA2* pathogenic variant carrier status and second contralateral breast, ovarian, and combined non-breast/ovarian cancer risks, adjusted for age and calendar year at breast cancer diagnosis, and estrogen receptor status of the first breast cancer diagnosis, and receipt of chemotherapy, radiotherapy, and hormonal therapy

	Number of females	Person Years	Events	HR[1] (95%CI[2])
Contralateral breast SPCs[3]				
Non-carriers	20035	70434	257	1.00 (reference category)
BRCA1[4] PV[5] carriers	1713	3737	57	3.69 (2.71-5.02)
BRCA2[6] PV carriers	1587	4015	38	2.47 (1.75-3.50)
Ovarian SPCs				
Non-carriers	20764	79470	30	1.00 (reference category)
BRCA1 PV carriers	1730	4325	40	34.1 (19.7-59.1)
BRCA2 PV carriers	1601	4169	20	12.3 (6.87-22.1)
Non-breast/ovarian SPCs				
Non-carriers	21543	87814	424	1.00 (reference category)
BRCA1 PV carriers	1840	7971	45	1.48 (1.07-2.04)
BRCA2 PV carriers	1750	8016	48	1.26 (0.93-1.70)

1: Hazard Ratio, 2: Confidence Interval, 3: Second Primary Cancer, 4: Breast Cancer gene 1, 5: Pathogenic Variant, 6: Breast Cancer gene 2, 7: Breast Cancer gene 1 and Breast Cancer gene 2.

Table S10: Associations between *BRCA1/BRCA2* pathogenic variant carrier status and second contralateral breast, ovarian, and combined non-breast/ovarian cancer risks, adjusted for age and calendar year at breast cancer diagnosis, and estrogen receptor status of the first breast cancer diagnosis, including females that tested negative for pathogenic variants in one gene following predictive testing and that were untested for a pathogenic variant in the other gene in the non-carrier reference group

	Number of females	Person Years	Events	HR[1] (95%CI[2])
Contralateral breast SPCs[3]				
Non-carriers	20163	70844	260	1.00 (reference category)
BRCA1[4] PV[5] carriers	1713	3737	57	3.58 (2.63-4.87)
BRCA2[6] PV carriers	1587	4015	38	2.39 (1.69-3.37)
Ovarian SPCs				
Non-carriers	20884	79866	30	1.00 (reference category)
BRCA1 PV carriers	1730	4325	40	33.3 (19.2-57.6)
BRCA2 PV carriers	1601	4169	20	12.1 (6.76-21.7)
Non-breast/ovarian SPCs				
Non-carriers	21677	88342	425	1.00 (reference category)
BRCA1 PV carriers	1840	7971	45	1.46 (1.06-2.02)
BRCA2 PV carriers	1750	8016	48	1.25 (0.93-1.69)

1: Hazard Ratio, 2: Confidence Interval, 3: Second Primary Cancer, 4: Breast Cancer gene 1, 5: Pathogenic Variant, 6: Breast Cancer gene 2, 7: Breast Cancer gene 1 and Breast Cancer gene 2.

Table S11: Annual incidence rates during a 5-year period, and associated statistics for second primary cancer risks, stratified by year of first breast cancer diagnosis

FU[1]	Total py[2]	N[3] BC[4] survivors	O[5]	Inc[6] (95% CI[7]) per 10,000py
**Contralateral breast SPCs[9] in *BRCA1*[10] PV[11] carriers**				
First BC dx[12]: < 2013	1417	540	29	205 (140-290)
First BC dx: >= 2013	1678	1173	15	89.4 (52.2-144)
**Contralateral breast SPCs in *BRCA2*[13] PV carriers**				
First BC dx: < 2013	1693	586	19	112 (69.8-172)
First BC dx: >= 2013	1556	1001	7	45 (20.1-88.3)
**Contralateral breast SPCs in *BRCA1/BRCA2* PV non-carriers**				
First BC dx: < 2013	22970	5890	101	44 (36.0-53.2)
First BC dx: >= 2013	34313	14055	101	29.4 (24.1-35.6)
**Ovarian SPCs in *BRCA1* PV carriers**				
First BC dx: < 2013	1423	576	19	134 (83.1-204)
First BC dx: >= 2013	2286	1154	19	83.1 (51.7-127)
**Ovarian SPCs in *BRCA2* PV carriers**				
First BC dx: < 2013	1618	613	11	68.0 (36.1-118)
First BC dx: >= 2013	1869	988	8	42.8 (20.2-80.8)
**Ovarian SPCs in *BRCA1/BRCA2* PV non-carriers**				
First BC dx: < 2013	25187	6303	15	5.96 (3.48-9.58)
First BC dx: >= 2013	39930	14461	9	2.25 (1.11-4.11)
**Non-breast/ovarian SPCs in *BRCA1* PV carriers**				
First BC dx: < 2013	2489	614	13	52.2 (29.3-86.8)
First BC dx: >= 2013	3455	1226	13	37.6 (21.1-62.5)
**Non-breast/ovarian SPCs in *BRCA2* PV carriers**				
First BC dx: < 2013	2831	674	18	63.6 (39.0-98.3)
First BC dx: >= 2013	3152	1076	16	50.8 (30.2-80.5)
**Non-breast/ovarian SPCs in *BRCA1/BRCA2* PV non-carriers**				
First BC dx: < 2013	28309	6753	139	49.1 (41.4-57.8)
First BC dx: >= 2013	42340	14790	161	38.0 (32.5-44.2)

1: Follow-Up time elapsed, 2: Person Years, 3: Number, 4: Breast Cancer, 5: Number of second primaries observed, 6: Incidence per 10,000 person-years, 7: Confidence Interval, 8: Cumulative Risk, 9: Second Primary Cancer, 10 : Breast Cancer gene 1, 11: Pathogenic Variant, 12: Diagnosis, 13: Breast Cancer gene 2

Table S12: Distribution of *BRCA1* and *BRCA2* tests in females provided by genetics laboratories

Genetic testing laboratory name	Number of subjects with a *BRCA1*[1] PV[2] test (% positive)	Time period of *BRCA1* tests reported by laboratory	Number of subjects with a *BRCA2*[3] PV test (% positive)	Time period of *BRCA2* tests reported by laboratory
Cambridge Genomics Laboratory	1691 (5.2)	2012 -– 2019	1691 (3.8)	2012 -– 2019
Great Ormond Street Genetics Laboratory	1300 (4.8)	2014 – 2019	1303 (2.6)	2014 – 2019
Leeds Genetics Laboratory	2378 (8.7)	2008 – 2019	2330 (7.7)	2008 – 2019
Liverpool Genetics Laboratory	7 (85.7)	2015 – 2019	19 (84.2)	2011 – 2019
London South Genomics Laboratory Hub	2881 (8.2)	1997 – 2019	2884 (6.0)	1997 – 2019
Manchester Genetics Laboratory	1752 (9.2)	2007 – 2019	1771 (9.9)	2007 – 2019
Newcastle Genetics Laboratory	1184 (8.6)	2006 – 2019	1233 (9.8)	2006 – 2019
North West London Hospitals Genetics Laboratory	433 (6.2)	2015 – 2018	432 (6.0)	2015 – 2018
Nottingham Genetics Laboratory	2079 (7.2)	2010 – 2019	2074 (7.1)	2010 – 2019
Oxford Genetics Laboratory	1755 (5.8)	2013 – 2019	1750 (6.2)	2013 – 2019
Royal Marsden Genetics Laboratory	1398 (3.6)	2013 – 2018	1407 (4.3)	2013 – 2018
Severn Pathology	597 (3.0)	2015 – 2019	496 (2.8)	2015 – 2019
Sheffield Diagnostic Genetics Service	955 (5.4)	2013 – 2019	909 (5.5)	2013 – 2019
St. George's University Hospital Laboratory	1459 (15.1)	1996 – 2019	1451 (13.0)	1996 – 2019
Wessex Regional Genetics Laboratory	1923 (7.1)	2009 – 2019	1944 (7.9)	2009 – 2019
West Midlands Regional Genetics Laboratory	3286 (7.1)	2000 – 2019	3307 (7.7)	2000 – 2019

1: Breast Cancer gene 1, 2: Pathogenic Variant, 3: Breast Cancer gene 2

Note: This table includes data on positive *BRCA1* and *BRCA2* PV tests from double heterozygotes, who were each assigned the 'Other' *BRCA1/BRCA2* PV classification (Supplementary Methods).

Table S13: Distribution of *BRCA1* and *BRCA2* tests in males provided by genetics laboratories

Genetic testing laboratory name	Number of subjects with a *BRCA1*[1] PV[2] test (% positive)	Time period of *BRCA1* tests reported by laboratory	Number of subjects with a *BRCA2*[3] PV test (% positive)	Time period of *BRCA2* tests reported by laboratory
Cambridge Genomics Laboratory	26 (3.8)	2013 – 2019	26 (19.2)	2013 – 2019
Great Ormond Street Genetics Laboratory	23 (0.0)	2017 – 2019	23 (0.0)	2017 – 2019
Leeds Genetics Laboratory	29 (0.0)	2008 – 2019	28 (10.7)	2008 – 2019
Liverpool Genetics Laboratory	0 (..)	..	0 (..)	..
London South Genomics Laboratory Hub	39 (0.0)	2006 – 2019	39 (25.6)	2006 – 2019
Manchester Genetics Laboratory	36 (0.0)	2012 – 2019	40 (15.0)	2008 – 2019
Newcastle Genetics Laboratory	23 (0.0)	2007 – 2019	24 (16.7)	2007 – 2019
North West London Hospitals Genetics Laboratory	6 (0.0)	2016 – 2018	6 (33.3)	2016 – 2018
Nottingham Genetics Laboratory	28 (3.6)	2011 – 2019	30 (23.3)	2011 – 2019
Oxford Genetics Laboratory	36 (0.0)	2013 – 2019	37 (13.5)	2013 – 2019
Royal Marsden Genetics Laboratory	18 (0.0)	2013 – 2018	18 (0.0)	2013 – 2018
Severn Pathology	13 (0.0)	2015 – 2019	13 (15.4)	2015 – 2019
Sheffield Diagnostic Genetics Service	13 (0.0)	2014 – 2019	13 (23.1)	2014 – 2019
St. George's University Hospital Laboratory	28 (7.1)	2004 – 2019	29 (20.7)	2000 – 2019
Wessex Regional Genetics Laboratory	33 (3.0)	2009 – 2019	34 (20.6)	2009 – 2019
West Midlands Regional Genetics Laboratory	115 (1.7)	2000 – 2019	117 (12.0)	2000 – 2019

1: Breast Cancer gene 1, 2: Pathogenic Variant, 3: Breast Cancer gene 2

Figure S1: Breast cancer diagnosis dates per year in females by *BRCA1* and *BRCA2* pathogenic variant carrier status

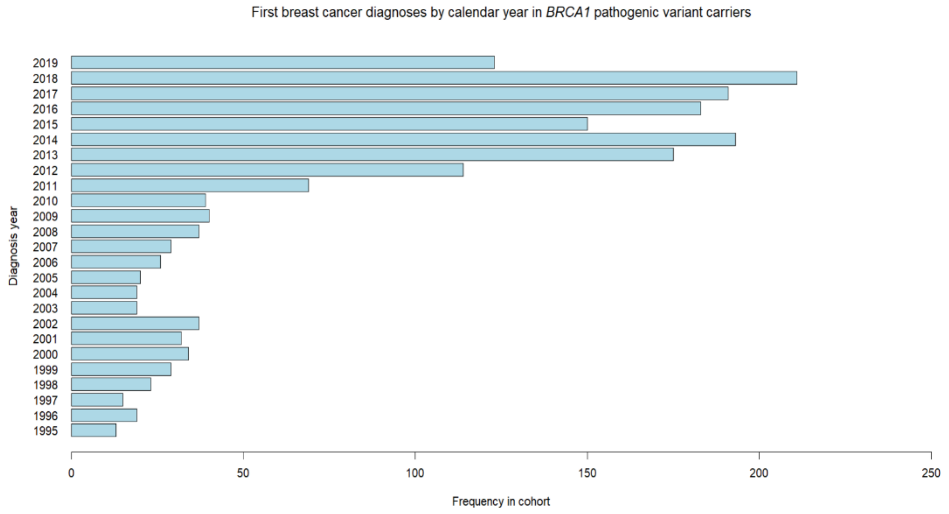

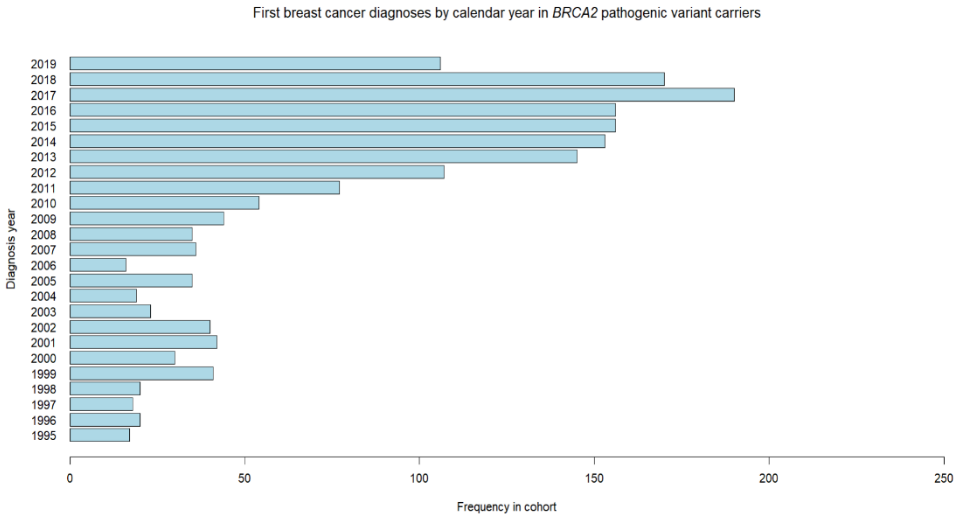

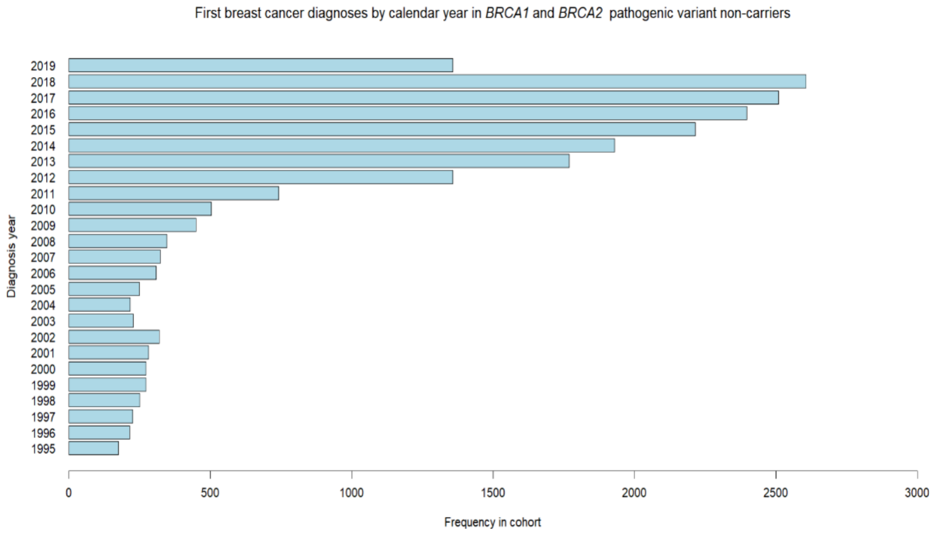

Figure S2: *BRCA1* test counts per year in females by *BRCA1* and *BRCA2* pathogenic variant carrier status

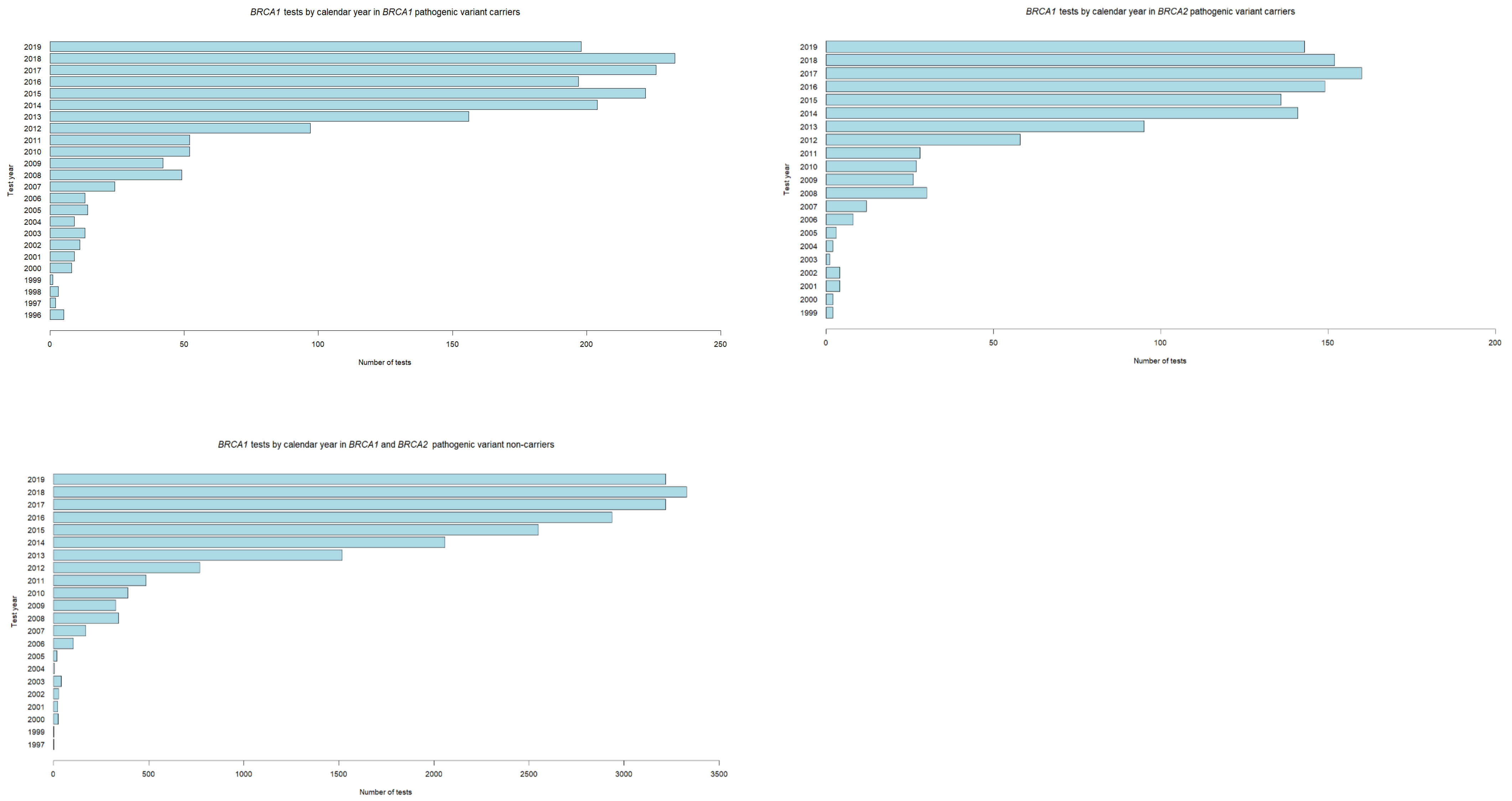

Figure S3: *BRCA2* test counts per year in females by *BRCA1* and *BRCA2* pathogenic variant carrier status

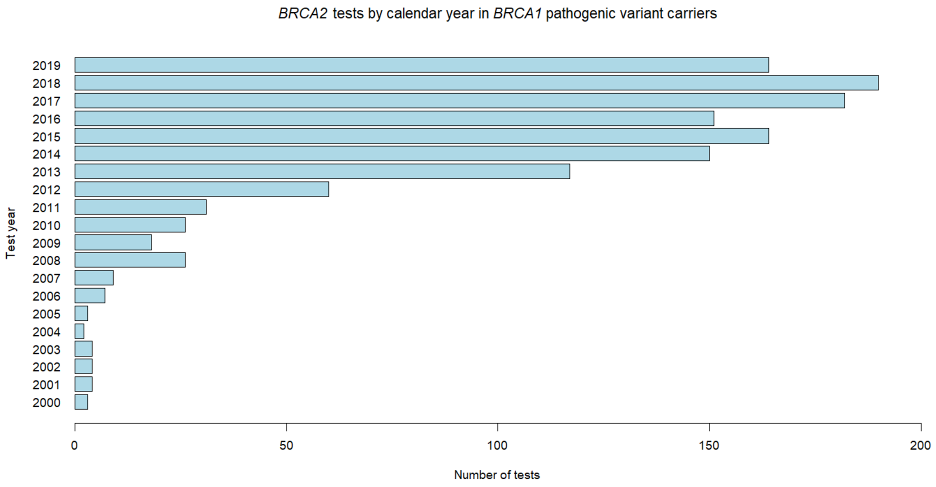

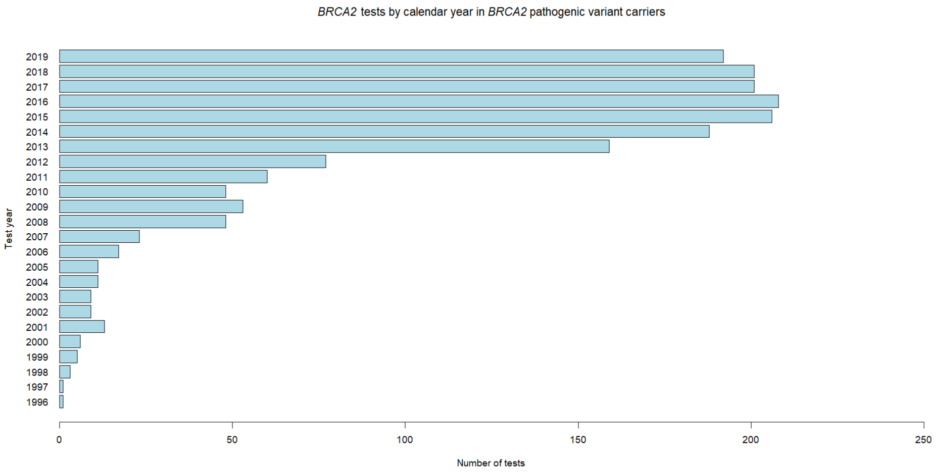

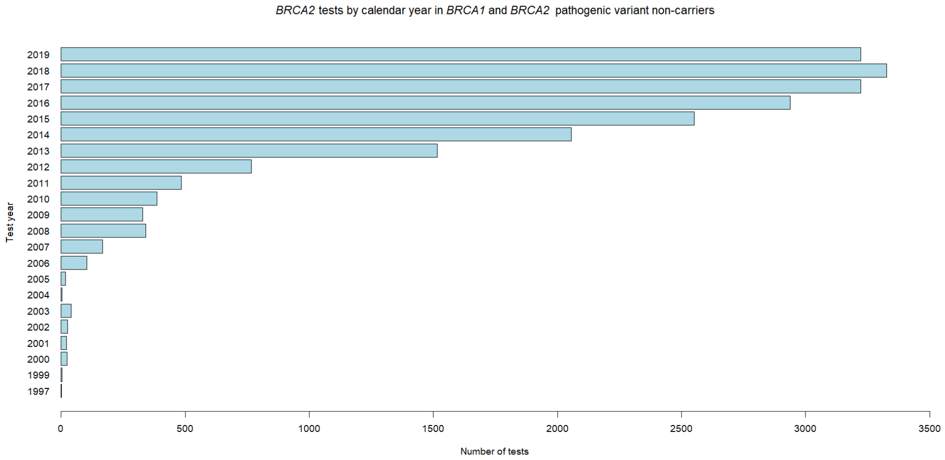

Figure S4: Follow-up time contributed by females, stratified by *BRCA1* and *BRCA2* pathogenic variant carrier status

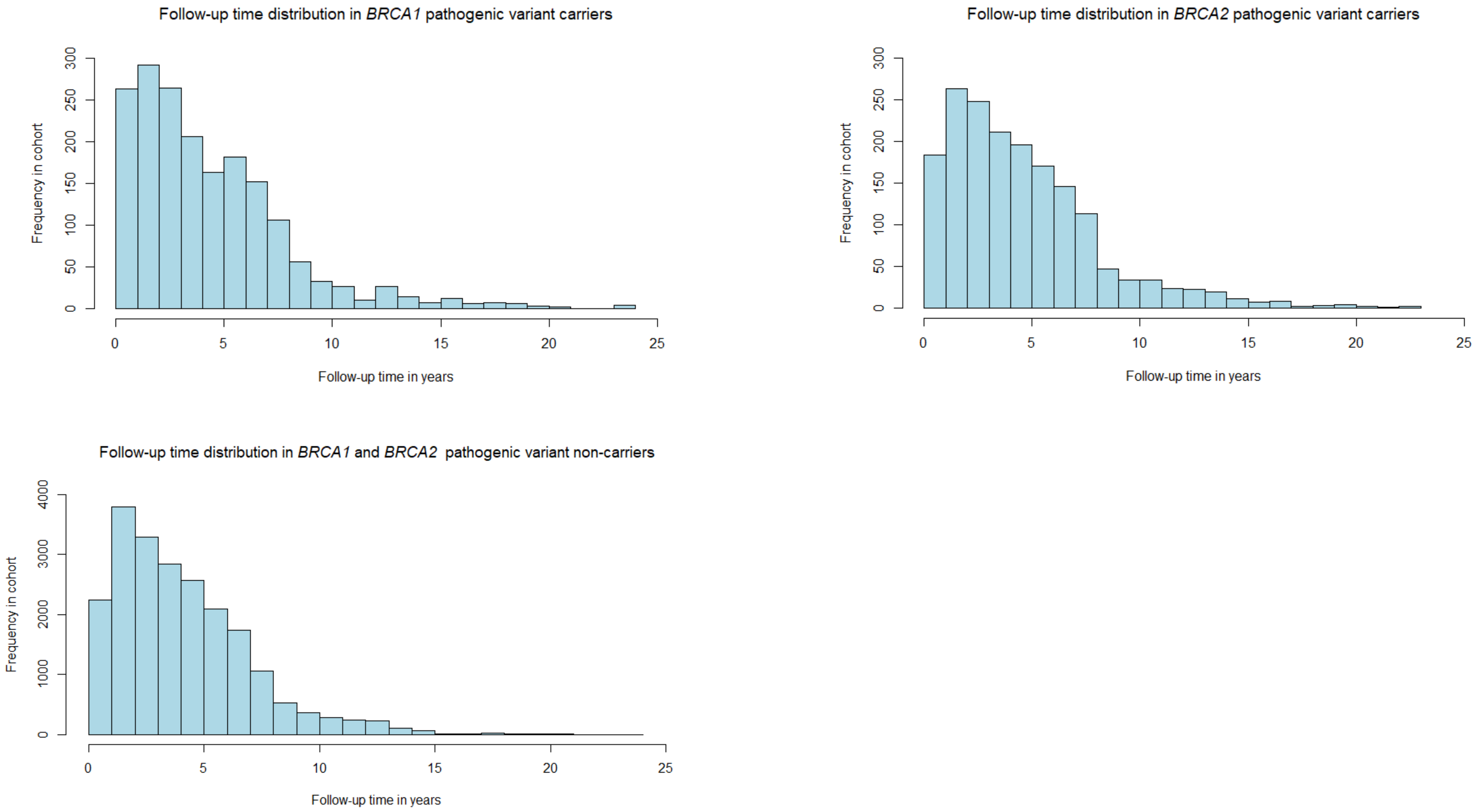

Figure S5: Breast cancer diagnosis dates per year in males by *BRCA1* and *BRCA2* pathogenic variant carrier status

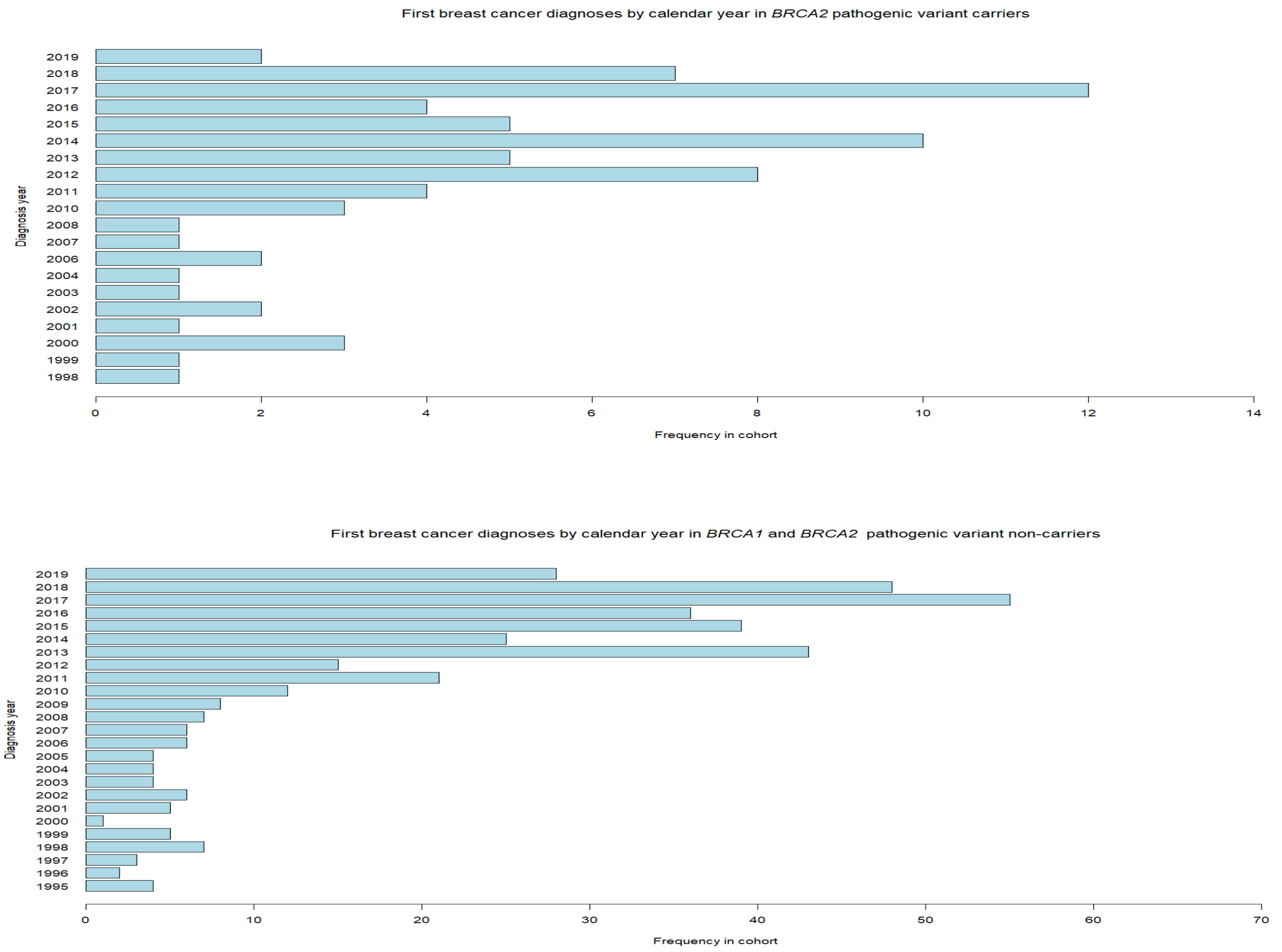

Figure S6: *BRCA1* test counts per year in males by *BRCA1* and *BRCA2* pathogenic variant carrier status

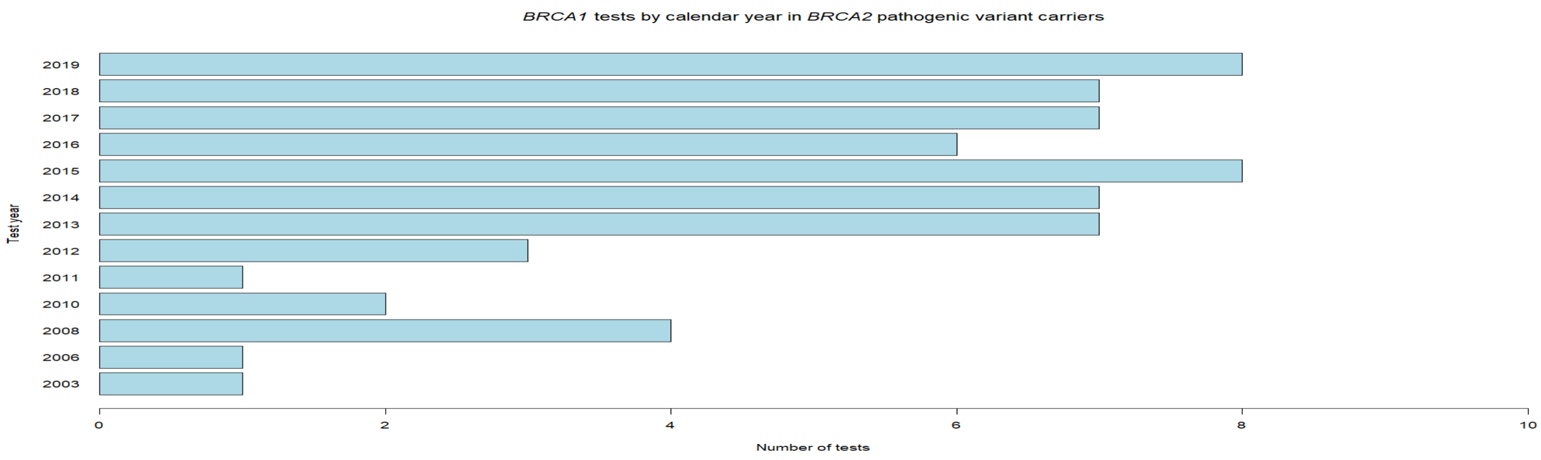

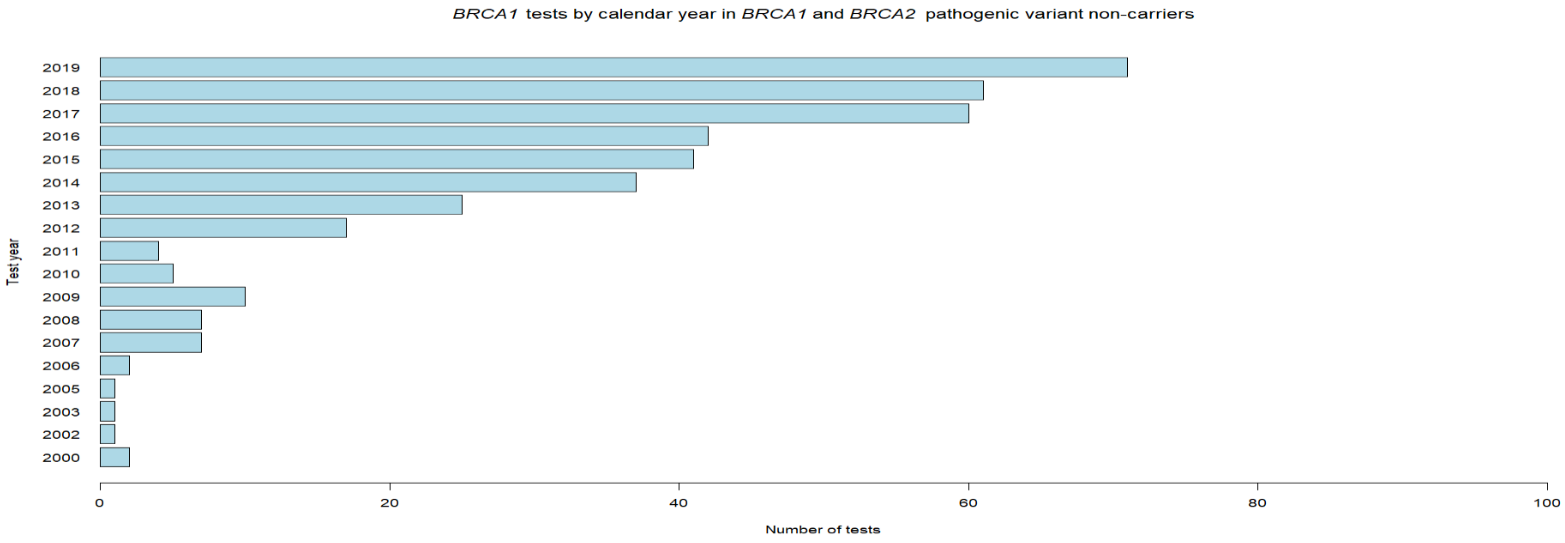

Figure S7: ***BRCA2*** **test counts per year in males by** ***BRCA1*** **and** ***BRCA2*** **pathogenic variant carrier status**

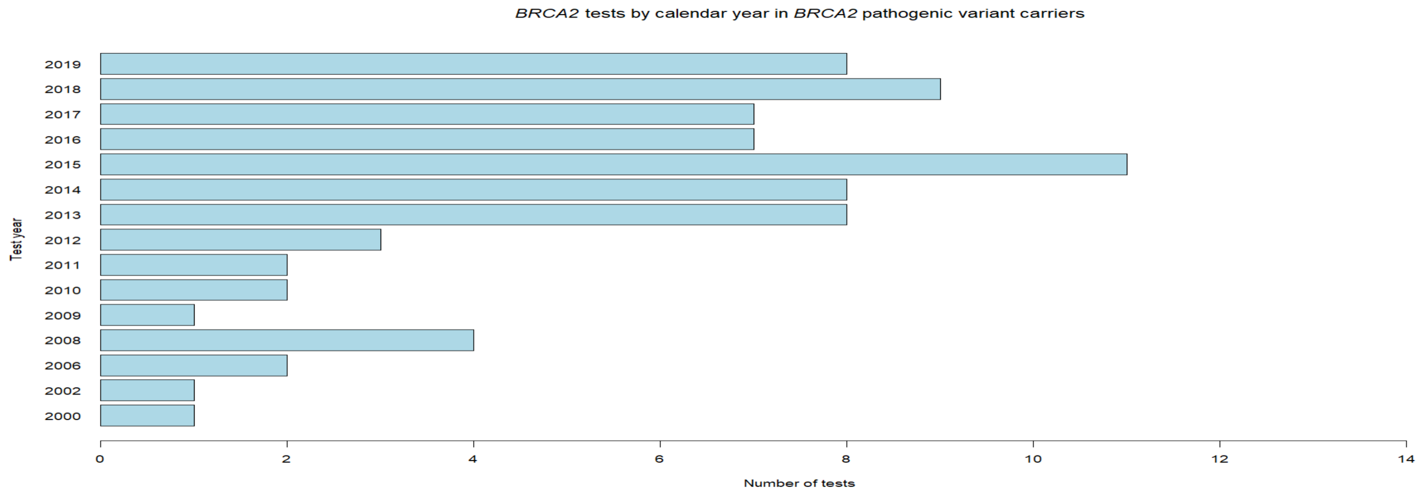

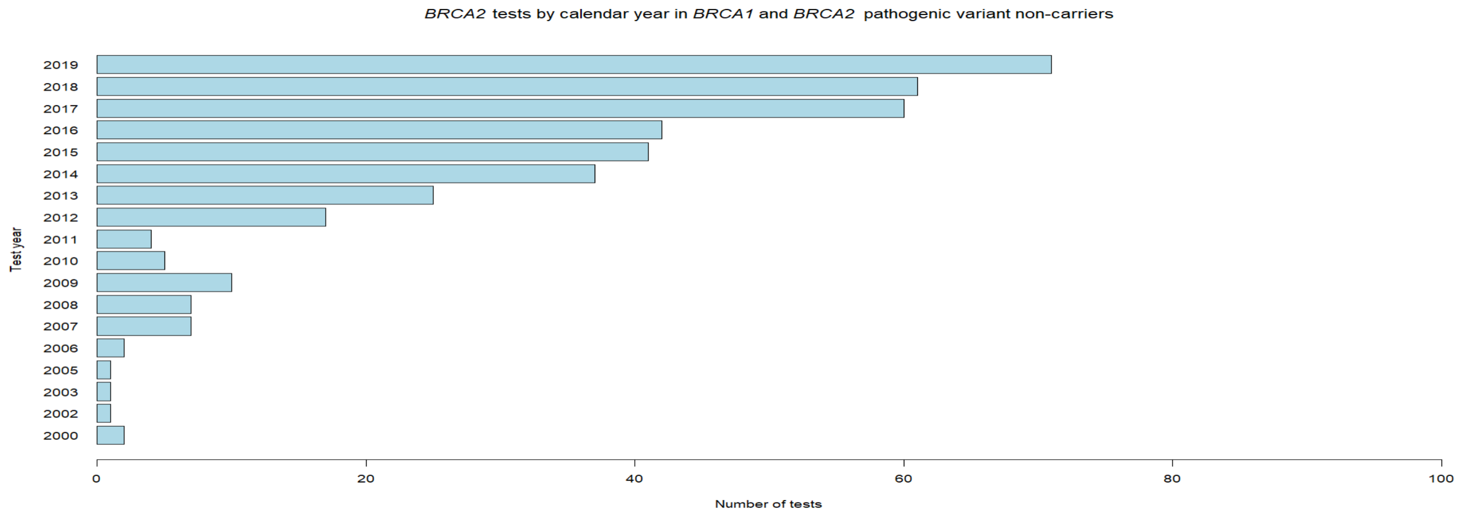

Figure S8: Follow-up time contributed by males, stratified by *BRCA1* and *BRCA2* pathogenic variant carrier status

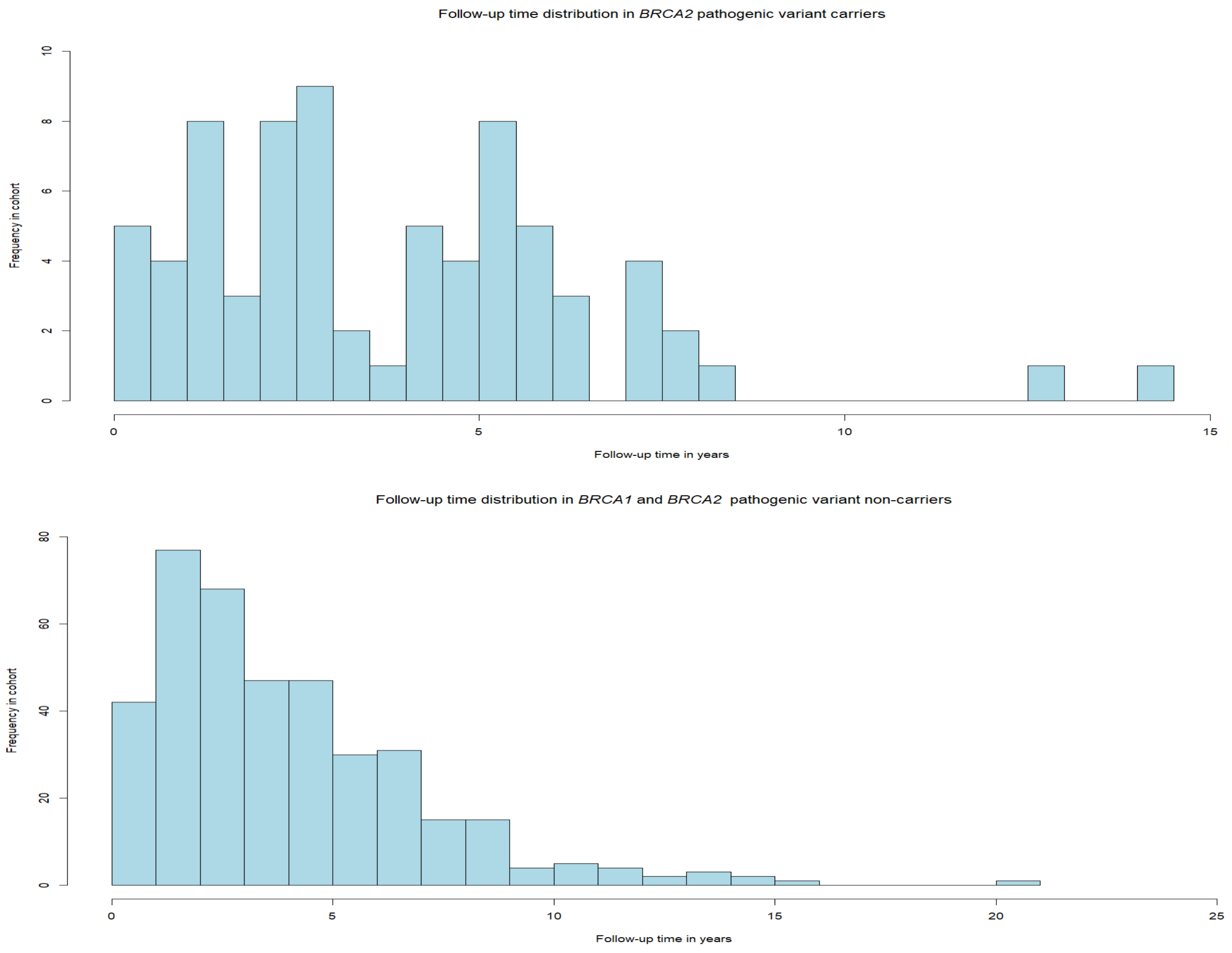

Figure S9: Logarithms of negative logarithms of survival functions against follow-up time for contralateral breast cancer development in females

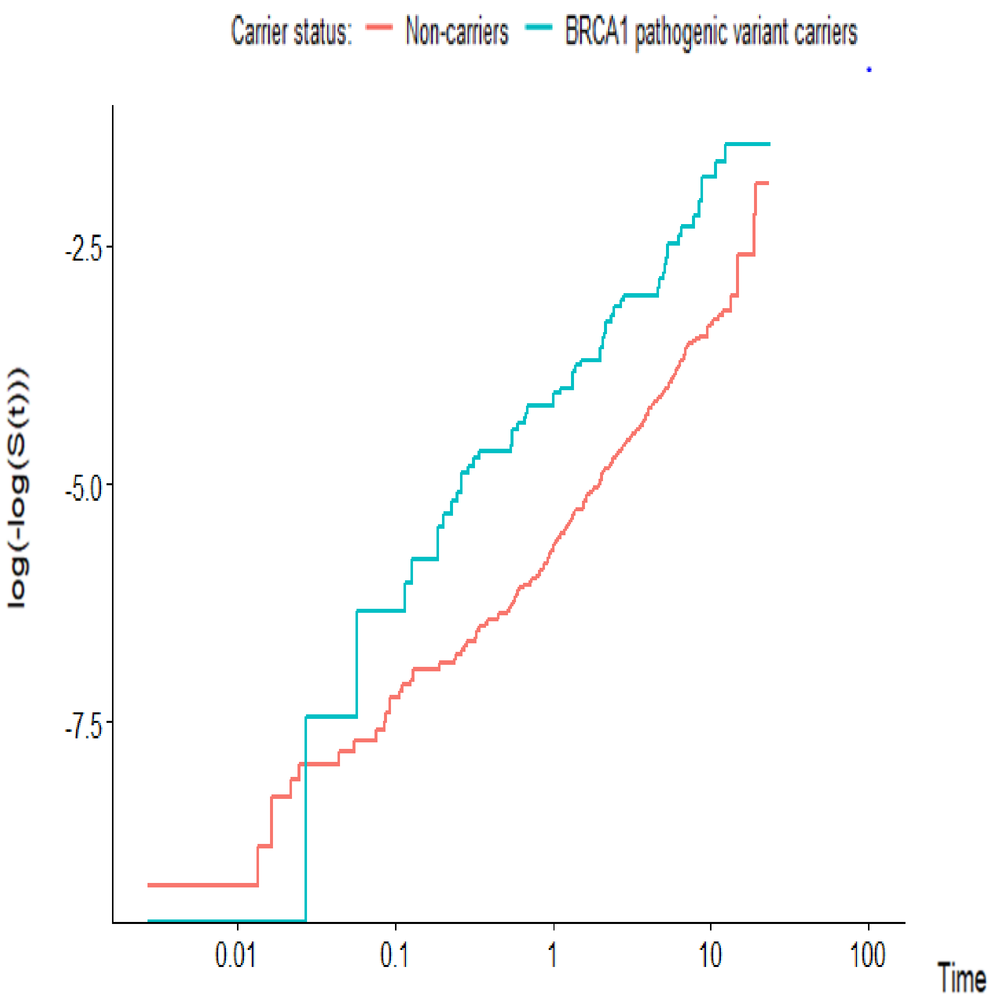

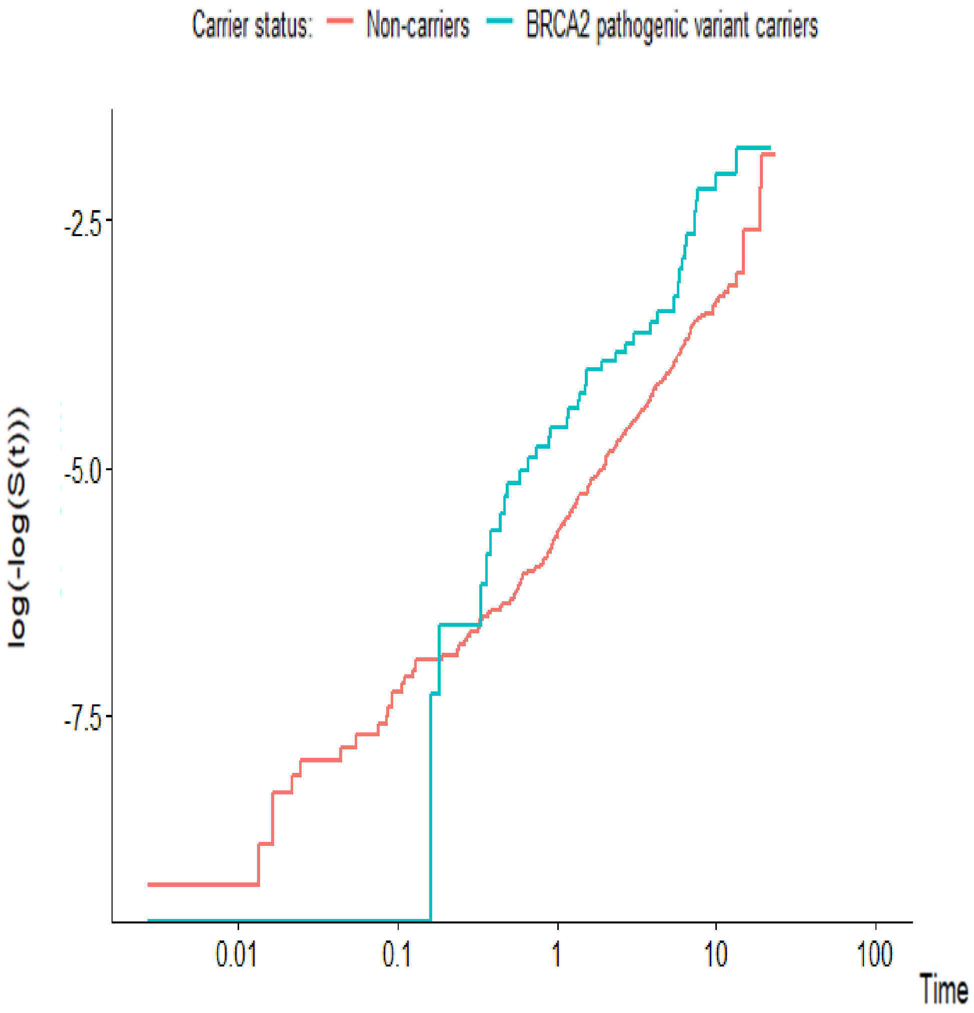

Figure S10: Logarithms of negative logarithms of survival functions against follow-up time for second primary ovarian cancer development

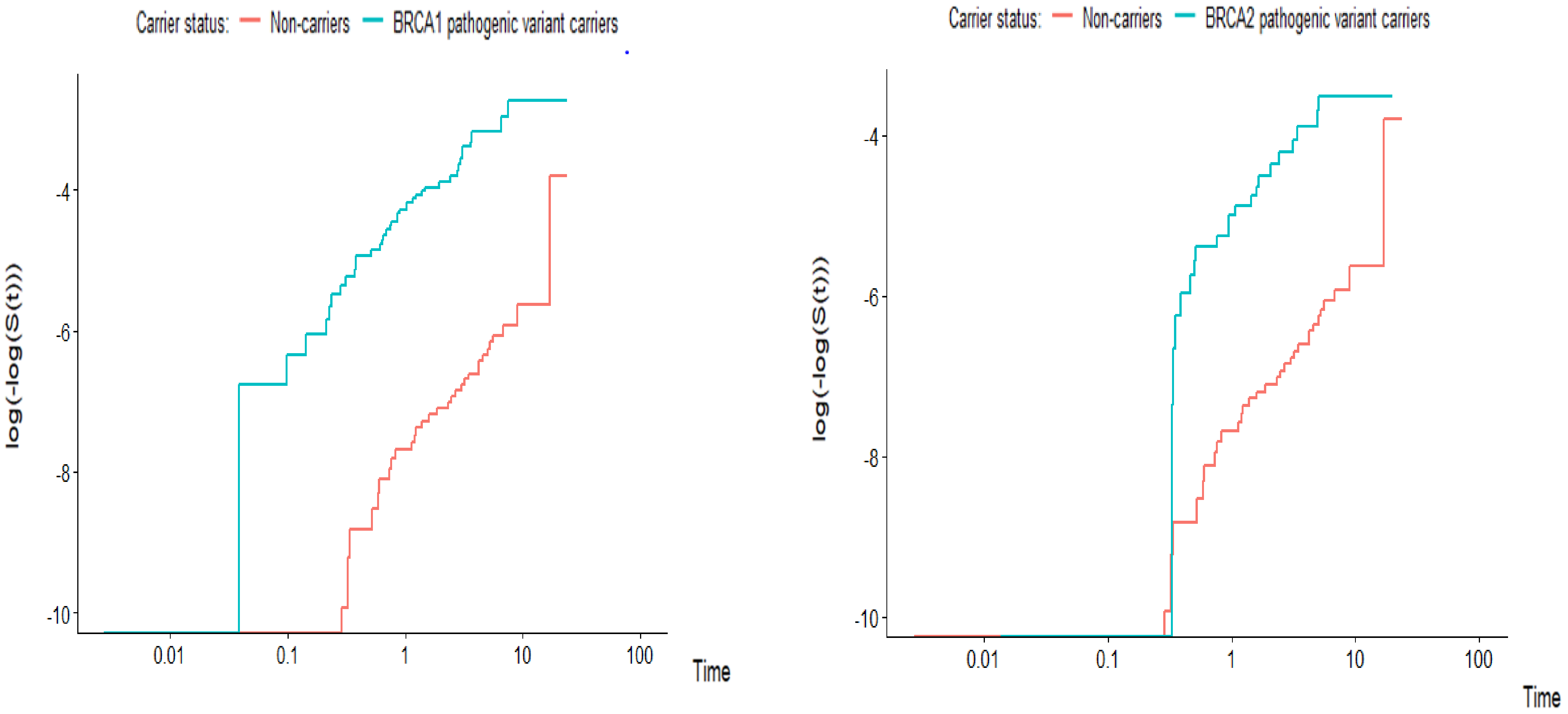

Figure S11: Logarithms of negative logarithms of survival functions against follow-up time for second primary non-breast and non-ovarian cancer development in females

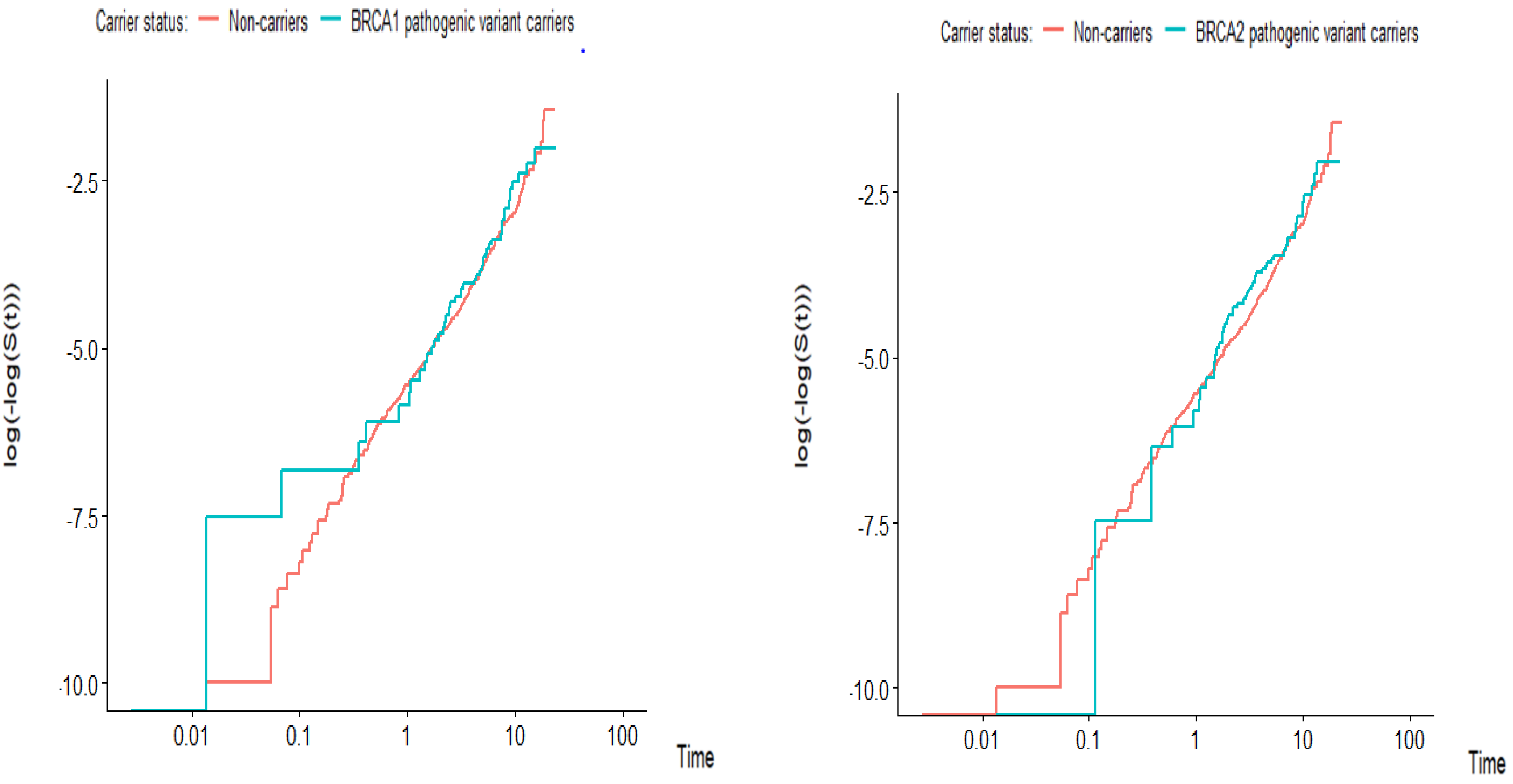

All abbreviations used and references made in the Data Supplement correspond to those used in the main text, with the exception of references prefaced with 'S'. These are supplementary references, and can be seen below.

Supplementary references

S1: Richards S, Aziz N, Bale S, et al. Standards and guidelines for the interpretation of sequence variants: a joint consensus recommendation of the American College of Medical Genetics and Genomics and the Association for Molecular Pathology. Genet Med 2015; **17:** 405-24.

S2: Garrett A, Durkie M, Callaway A, et al. Combining evidence for and against pathogenicity for variants in cancer susceptibility genes: CanVIG-UK consensus recommendations. J Med Genet 2021; **58:** 297-304.

S3: Karczewski, K.J., Francioli, L.C., Tiao, G. et al. Variation across 141,456 human exomes and genomes reveals the spectrum of loss-of-function intolerance across human protein-coding genes. PREPRINT [Accessed April 2024]. Available at https://www.biorxiv.org/content/10.1101/531210v3

S4: Landrum MJ, Lee JM, Riley GR, et al. ClinVar: public archive of relationships among sequence variation and human phenotype. Nucleic Acids Res. 2014; **42:** D980-5.

S5: Loong L, Cubuk C, Choi S, et al. Quantifying prediction of pathogenicity for within-codon concordance (PM5) using 7541 functional classifications of BRCA1 and MSH2 missense variants. Genet Med. 2022; **24:** 552-563.

S6: Jaganathan K, Kyriazopoulou Panagiotopoulou S, et al. Predicting Splicing from Primary Sequence with Deep Learning. Cell. 2019; **176:** 535-548.e24.

S7: Ioannidis NM, Rothstein JH, Pejaver V, et al. REVEL: An Ensemble Method for Predicting the Pathogenicity of Rare Missense Variants. Am J Hum Genet. 2016; **99:** 877-885.

S8: Wai HA, Lord J, Lyon M, et al. Blood RNA analysis can increase clinical diagnostic rate and resolve variants of uncertain significance. Genet Med. 2020; **22:** 1005-1014.

S9: Walker LC, Whiley PJ, Couch FJ, et al. Detection of splicing aberrations caused by BRCA1 and BRCA2 sequence variants encoding missense substitutions: implications for prediction of pathogenicity. Hum Mutat. 2010; **31:** E1484-505.

S10: Houdayer C, Caux-Moncoutier V, Krieger S, et al. Guidelines for splicing analysis in molecular diagnosis derived from a set of 327 combined in silico/in vitro studies on BRCA1 and BRCA2 variants. Hum Mutat. 2012; **33:** 1228-38.

S11: Ikegami M, Kohsaka S, Ueno T, et al. High-throughput functional evaluation of BRCA2 variants of unknown significance. Nat Commun. 2020; **11:** 2573.

S12: Richardson ME, Hu C, Lee KY, et al. Strong functional data for pathogenicity or neutrality classify BRCA2 DNA-binding-domain variants of uncertain significance. Am J Hum Genet. 2021; **108:** 458-468.

S13: Mesman RLS, Calléja FMGR, Hendriks G, et al. The functional impact of variants of uncertain significance in BRCA2. Genet Med. 2019; **21:** 293-302.

S14: Hart SN, Hoskin T, Shimelis H, et al. Comprehensive annotation of BRCA1 and BRCA2 missense variants by functionally validated sequence-based computational prediction models. Genet Med. 2019; **21:** 71-80.

S15: Guidugli L, Shimelis H, Masica DL, et al. Assessment of the Clinical Relevance of BRCA2 Missense Variants by Functional and Computational Approaches. Am J Hum Genet. 2018; **102:** 233-248.

S16: Starita LM, Islam MM, Banerjee T, et al. A Multiplex Homology-Directed DNA Repair Assay Reveals the Impact of More Than 1,000 BRCA1 Missense Substitution Variants on Protein Function. Am J Hum Genet. 2018; **103:** 498-508.

S17: Petitalot A, Dardillac E, Jacquet E, et al. Combining Homologous Recombination and Phosphopeptide-binding Data to Predict the Impact of BRCA1 BRCT Variants on Cancer Risk. Mol Cancer Res. 2019; **17:** 54-69.

S18: Fernandes VC, Golubeva VA, Di Pietro G, et al. Impact of amino acid substitutions at secondary structures in the BRCT domains of the tumor suppressor BRCA1: Implications for clinical annotation. J Biol Chem. 2019; **294:** 5980-5992.

S19: Findlay GM, Daza RM, Martin B, et al. Accurate classification of BRCA1 variants with saturation genome editing. Nature. 2018; 562: **217-222.**

S20: Bouwman P, van der Heijden I, van der Gulden H, et al. Functional Categorization of BRCA1 Variants of Uncertain Clinical Significance in Homologous Recombination Repair Complementation Assays. Clin Cancer Res. 2020; **26:** 4559-4568.

S21: Easton DF, Deffenbaugh AM, Pruss D, et al. A systematic genetic assessment of 1,433 sequence variants of unknown clinical significance in the BRCA1 and BRCA2 breast cancer-predisposition genes. Am J Hum Genet. 2007; **81:** 873-83.

S22: Parsons MT, Tudini E, Li H, et al. Large scale multifactorial likelihood quantitative analysis of BRCA1 and BRCA2 variants: An ENIGMA resource to support clinical variant classification. Hum Mutat. 2019; **40:** 1557-1578.

S23: Sherry ST, Ward M, Sirotkin K. dbSNP-database for single nucleotide polymorphisms and other classes of minor genetic variation. Genome Res. 1999; **9:** 677-9.

S24: Kopanos C, Tsiolkas V, Kouris A, et al. VarSome: the human genomic variant search engine. Bioinformatics. 2019; **35:** 1978-1980.

S25: Heidemann S, Fischer C, Engel C, et al. Double heterozygosity for mutations in BRCA1 and BRCA2 in German breast cancer patients: implications on test strategies and clinical management. Breast Cancer Res Treat 2012; **134:** 1229-39.

S26: White IR, Royston P. Imputing missing covariate values for the Cox model. *Stat Med.* 2009; **28(15):** 1982-98.

S27: Prat J; FIGO Committee on Gynecologic Oncology. Staging classification for cancer of the ovary, fallopian tube, and peritoneum. Int J Gynaecol Obstet 2014; **124:** 1-5.

S28: Kalsi JK, Ryan A, Gentry-Maharaj A, et al. Completeness and accuracy of national cancer and death registration for outcome ascertainment in trials-an ovarian cancer exemplar. Trials 2021; **22:** 88.

S29: Dowle M, Srinivasan A (2023). data.table: Extension of ‘data.frame’. R package version 1.14.8, <https://CRAN.R-project.org/package=data.table>

S30: R Special Interest Group on Databases (R-SIG-DB), Hadley Wickham and Kirill Müller (2021). DBI: R Database Interface. R package version 1.1.2, <https://CRAN.R-project.org/package=DBI>

S31: Hadley Wickham, Romain François, Lionel Henry and Kirill Müller (2021). dplyr: A Grammar of Data Manipulation. R package version 1.0.7, <https://CRAN.R-project.org/package=dplyr>

S32: Stevenson M, Sargeant E (2023). epiR: Tools for the Analysis of Epidemiological Data. R package version 2.0.62, <https://CRAN.R-project.org/package=epiR>

S33: Hadley Wickham (2021). forcats: Tools for Working with Categorical Variables (Factors). R package version 0.5.1, <https://CRAN.R-project.org/package=forcats>

S34: Schmidt D, Chen W (2017). “getPass: Masked User Input.” R package version 0.2-2, < https://CRAN.R-project.org/package=getPass>

S35: Stef van Buuren, Karin Groothuis-Oudshoorn (2011). mice: Multiple Imputation by Chained Equations in R. Journal of Statistical Software, 45(3), 1-67. DOI 10.18637/jss.v045.io3.

S36: Garrett Grolemund, Hadley Wickham (2011). Dates and Times Made Easy with lubridate. Journal of Statistical Software, 40(3), 1-25. URL https://www.jstatsoft.org/v40/i03/.

S37: Hadley Wickham and Jennifer Bryan (2022). readxl: Read Excel Files. R package version 1.4.0, <https://CRAN.R-project.org/package=readxl>

S38: Urbanek S (2021). rJava: Low-Level R to Java Interface. R package version 1.0-6, <https://CRAN.R-project.org/package=rJava>

S39: Urbanek S (2022). RJDBC: Provides Access to Databases Through the JDBC Interface. R package version 0.2-10, <https://CRAN.R-project.org/package=RJDBC>

S40: Therneau T (2021). A Package for Survival Analysis in R. R package version 3.2-13, <https://CRAN.R-project.org/package=survival>.

S41: Alboukadel Kassambara, Marcin Kosinski and Przemyslaw Biecek (2021). survminer: Drawing Survival Curves using 'ggplot2'. R package version 0.4.9, <https://CRAN.R-project.org/package=survminer>

S42: Grosjean, Ph. (2023). SciViews::R. UMONS, Mons, Belgium. URL: https://sciviews.r-universe.dev/.
