# Supplementary material for: Second Primary Cancer Risks After Breast Cancer in BRCA1 and BRCA2 Pathogenic Variant Carriers
Source: J Clin Oncol. Author manuscript; Available in PMC 2025 Feb 20. (PMC7616773; doi:10.1200/JCO.24.01146)
Supplement: Supplementary file [file EMS199754-supplement-Supplementary_file.pdf]

ASCO Journals Data Sharing Statement Questionnaire

**1. Manuscript Title**	Second primary cancer risks following breast cancer in BRCA1 and BRCA2 pathogenic variant carriers
**2. First Author Last Name**	Allen
**3. Does your manuscript use ONLY data from a publicly available database (eg, SEER, Medicare)?**<br>If "Yes," please specify the database; no additional information is needed.<br>If "No," please complete questions 4-17.	no
**4. Will the data collected for your study be made available to others?**<br>(If "No," enter context for your decision)	This work uses data provided by patients and collected by the National Health Service (NHS) as part of their care and support. The data are collated, maintained and quality assured by the Nartional Disease Registration Service, which is part of NHS England. Data in this manuscript may be accessed through application to NHS England.
**5. List which data are available**	All data can be accessed through above application
**6. Additional information about the data**<br>(Enter "None," if applicable)	None
**7. List how or where the data can be obtained**<br>(e.g., e-mail address, URL, or other repository)	Application to NHS England
**8. List beginning and ends dates that data will be available**<br>(e.g., MM/DD/YYYY to MM/DD/YYYY)	Data in manuscript : 1995-2020. Other years available through application to NHS England
**9. List any supporting documents**<br>(Enter "None," if applicable)	None
**10. Enter additional information about supporting documents**<br>(Enter "None," if applicable)	Not applicable

11. **How or where can supporting documents be obtained?**<br>(e.g., e-mail address, URL, or other repository)	Not applicable
12. **List beginning and ends dates that supporting documents will be available**<br>(e.g., MM/DD/YYYY to MM/DD/YYYY)	Not applicable
13. **Indicate to whom data will be available** (Enter "U/K" for unknown; "N/A" for not applicable)	Those that have an application to NHS England approved
14. **Indicate for what type of analysis or purpose**<br>(Enter "U/K" for unknown)	At discretion of applicant - purpose will need to be stated at application
15. **Indicate by what mechanism**<br>(Enter "U/K" for unknown)	Made available within NHS England environment following establishment of login credentials, etc
16. **Enter any other restrictions**<br>(Enter "None," if applicable)	None
17. **Enter any additional information**<br>(Enter "None," if applicable)	None
